# Inhibition of Cathepsin S Induces Mitochondrial Apoptosis in Glioblastoma Cell Lines Through Mitochondrial Stress and Autophagosome Accumulation

**DOI:** 10.3389/fonc.2020.516746

**Published:** 2020-12-23

**Authors:** Maoxing Fei, Li Zhang, Handong Wang, Yihao Zhu, Wenhao Niu, Ting Tang, Yanling Han

**Affiliations:** ^1^ Department of Neurosurgery, Jinling Hospital, Nanjing Medical University, Nanjing, China; ^2^ Department of Neurosurgery, Jinling Hospital, Medical School of Nanjing University, Nanjing, China; ^3^ Department of Neurosurgery, Jinling Hospital, School of Medicine, Southeast University, Nanjing, China; ^4^ Department of Neurosurgery, Jinling Hospital, Nanjing, China

**Keywords:** cathepsin S, glioblastoma, autophagy, mitophagy, mitochondrial calcium uniporter

## Abstract

Cathepsin S (CTSS), a lysosomal cysteine protease, is overexpressed in various cancers, including glioblastoma (GB). A high level of CTSS is associated with tumor progression and poor outcome in GB. However, the underlying mechanisms of its role in the biological characteristics of G5B remain to be elucidated. Here, we uncovered a potential role of CTSS in the lysosomes and mitochondria of GB cells (GBCs). Downregulation of CTSS in GBCs could increase the expression of autophagy-related proteins; however, there was no significant change in p62, suggesting autophagy blockade. Moreover, inhibition of CTSS increased the expression of mitochondrial calcium uniporter (MCU) and enhanced mitochondrial Ca^2+^ uptake ability, causing mitochondrial Ca^2+^ overload, the generation of copious reactive oxygen species (ROS) and eventual mitochondrial apoptosis. Additionally, elevated damage to mitochondria exacerbated the burden of autophagy. Finally, we found that silence of MCU could alleviate the inhibition of CTSS-induced autophagosome accumulation and mitochondrial stress. Collectively, these results demonstrate that CTSS plays an important role in the process of autophagic flux and mitochondrial functions in GBCs.

## Introduction

Glioma is the most common malignant brain cancer (1). Glioblastoma (GB) (or glioblastoma multiforme), which accounts for approximately 15% of all primary brain and central nervous system tumors and 55% of all gliomas, is regarded as the most aggressive and fatal type of glioma (2). Due to the high progression and invasion capabilities and therapeutic resistance of GBs, patients with GBs have a 5-year survival rate of 5% (2). Even when massive or total resection of the tumor is applied along with radiotherapy and chemotherapy, the prognosis of GB patients remains poor, with a median survival time of approximately 9–12 months (3). Therefore, effective therapeutic strategies to improve GB patient prognosis should be developed.

Cathepsin S (CTSS), a primarily lysosomal enzyme that can also be found outside the lysosome, has a series of functions under extracellular conditions, unlike other family members (4). It serves as a pivotal mediator of antigen presentation in major histocompatibility complex class II (5). Since CTSS can be transferred to the extracellular matrix (ECM), it degrades diverse ECM proteins, including laminin, fibronectin, elastin, osteocalcin, and some collagens, thus promoting tumor invasion and metastasis (6). CTSS is also highly expressed in various tumor tissues, including GB (7). Our previous study showed that CTSS inhibitors could induce autophagy and apoptosis in GB cells (GBCs) through reactive oxygen species (ROS)-mediated PI3K/AKT/mTOR/p70S6K and JNK signaling pathways (8). However, how ROS are generated after CTSS inhibition still requires investigation.

ROS are generated mostly by mitochondria. Previous studies indicated that CTSS could influence intracellular Ca^2+^ levels in tumor cells (9). While optimal mitochondrial function depend on adequate Ca^2+^ levels, erratic Ca^2+^ oscillations compromise mitochondrial bioenergetics (10). Under physiological conditions, the maintenance of adequate Ca^2+^ levels in the mitochondrial matrix is required for the mitochondrial tricarboxylic acid (TCA) cycle, as Ca^2+^ is an essential cofactor for many TCA cycle-related enzymes, such as pyruvate dehydrogenase, isocitrate dehydrogenase, and α-ketoglutarate dehydrogenase (11). However, during pathological events, mitochondrial Ca^2+^ overload is lethal to tumor cells (11). Ca^2+^ overload influences PTP, decreases mitochondrial membrane potential (MMP), compromises oxidative phosphorylation and generates copious amounts of ROS (11).

Ca^2+^ plays various roles in cell physiology (12, 13). Due to its functions under normal conditions and because it does not degrade, Ca^2+^ appears difficult to target in tumor studies. However, subtle changes in Ca^2+^ levels under different conditions can cause enormous functional changes and affect some important pro- and antitumor transcription factors (11, 14). In fact, cancers can manipulate Ca^2+^ signals to achieve progression, migration, and metastasis and gain treatment resistance (15, 16). In addition, studies have shown that some Ca^2+^ transport proteins can be oncogenic or even cause tumors to adopt more malignant phenotypes (17–20). Targeting some important Ca^2+^ transport proteins would be lethal to cancer cells (11, 17, 21, 22). Ca^2+^ accumulation in mitochondria can generate more ROS and cause cell death (23). Mitochondria calcium uniporter (MCU) complex, a pivotal mitochondrial Ca^2+^ uptake protein complex, was discovered relatively recently compared with other mitochondrial Ca^2+^ channels (24); however, its role in the mitochondrial Ca^2+^ balance has received increasing attention (25, 26). Here, we found that MCU-mediated mitochondrial Ca^2+^ influx plays a novel role in regulating autophagic flux and mitochondrial apoptosis in CTSS-targeted GBCs.

## Materials and Methods

### Clinical Samples and Cell Lines

GB tissue samples were collected from Jinling Hospital. Paired adjacent non-tumor tissues were isolated from the tumor border and were shown to lack tumor cells by microscopy. This study was performed in accordance with the Declaration of Helsinki and approved by the Institutional Review Board, Nanjing University. The consent obtained from the participants was both informed and written.

The U87, U251, and A172 human GBC lines were provided by the Cell Bank of Type Culture Collection of the Chinese Academy of Sciences (Shanghai, China). Human astrocytes (HAs) were obtained from the Institute of Basic Medical Sciences (Beijing, China). GBM cell lines were cultured in DMEM containing 1% penicillin/streptomycin (HyClone, GE Healthcare Life Sciences, Logan, UT, USA) and 10% fetal bovine serum (Thermo Fisher Scientific). HAs were grown in astrocyte medium (AM, ScienCell, San Diego, CA, USA) as instructed by the company. All cells were incubated at 37°C in a humidified incubator containing 5% CO_2_.

### Cell Transfection

To develop stable CTSS knockdown cells, lentivirus carrying shRNA targeting human CTSS and a negative control lentivirus were designed and generated by GeneChem Co., Ltd. (Shanghai, China). To knock down *MCU* expression in U251 and U87 cell lines, siRNA targeting MCU was designed and generated by GeneChem Co., Ltd. The primer sequence of *CTSS* shRNA: 5’-CCACAATTTGGTGAAGAA-3’. The primer sequence of *MCU* siRNA:5’GCCAGAGACAGACAAUACUtt-3’, 3’-ttCGGUCUCUGUCUGUUAUGA-5’. Cells were transfected with the lentivirus according to the company’s instructions. The knockdown efficiency was detected via western blotting.

### Reagents and Antibodies

The selective CTSS inhibitor Z-FL-COCHO (ZFL) was purchased from Calbiochem Co. (Darmstadt, Germany). Temozolomide (TMZ, 76899) and BafilomycinA1 (BafA1) was purchased from Sigma-Aldrich (Missouri, USA). BAPTA-AM was purchased from Selleck Chemicals (Shanghai, China). Anti-Bax (#5023), anti-Bcl-2 (#3498), anti-cleaved caspase 3 (#9661), anti-PARP (#9532), anti-Atg3 (#3415), anti-Atg5 (#9980), anti-Atg7 (#2631), anti-Atg12 (#2010), and anti-β-actin (#4970) antibodies were purchased from Cell Signaling Technology (Danvers, MA, USA). Anti-LC3II (NB100-2220) and anti-Beclin 1 (NB110-87318) antibodies were purchased from Novus Biologicals (Littleton, CO, USA). Anti-CTSS (ab134157), anti-CD31 (ab28364), anti-MCU (ab121499), anti-MICU1 (ab190114), anti-MICU2 (ab101465), anti-EMRE (ab122209), and anti-p62/SQSTM1 (ab91526) antibodies were purchased from Abcam (Cambridge, MA, USA). Anti-Mff (17090-1-AP), anti-Drp1 (12957-1-AP), and anti-OPA1 (27733-1-AP) antibodies were purchased from Proteintech Inc(Rosemont, MA, USA).

### Cell Viability Assay

Cell viability was detected by Cell Counting Kit-8 (CCK-8) assay (Dojindo, Kumamoto, Japan) according to the manufacturer’s instructions. Briefly, the assay was carried out as follows: Cells were plated in 96-well plates at a density of 5 × 10^3^ cells/well for 12 h. After treatment, cells were incubated with 100 µL DMEM containing 10% CCK-8 reagent in a 37°C incubator. Two hours later, optical density values were detected with an enzyme-linked immunosorbent assay plate reader (Bio-Rad Laboratories, Inc., Berkeley, CA). Then, the following formula was used to calculate the viability: cell viability = (OD_450_ of treated group/OD_450_ of control group) × 100%.

### Flow Cytometric Analysis

To analyze apoptosis, transfected cells were harvested, and an Annexin V-APC/PI apoptosis detection kit (C1062M, Beyotime Co) was used as instructed in the manufacturer’s protocol. Then, apoptotic cells were analyzed with a FACS Calibur ﬂow cytometer (BD Biosciences, New Jersey).

To analyze the intracellular and mitochondrial Ca^2+^ content, Fluo-2 (KGAF022, KeyGEN Co., Nanjing), and Rhod-2/AM (#40776ES50, Yeasen Co., Shanghai) were diluted to recommended concentration with 10% FBA culture medium and loaded into the transfected cells for 60 min at 25°C. Finally, the fluorescence intensity was detected by flow cytometry (BD Biosciences) and imaged with a Zeiss immunoﬂuorescence microscope (Zeiss, Germany).

Total intracellular ROS levels were determined by staining cells with dichlorofluorescin diacetate (DCFH-DA, S0033, Beyotime). Briefly, cells were washed with PBS and incubated with 10 μM DCFH-DA at 37°C for 30 min. Cells were then washed twice with PBS and analyzed by flow cytometry (BD Biosciences).

### Western Blot Analysis

Western blot analysis was carried out as previously described (8). Briefly, proteins were resolved by SDS-PAGE and then transferred onto PVDF membranes (EMD Millipore, Billerica, MA, USA). After blocking with 5% skim milk, the membranes were incubated with primary antibodies (1/1000) followed by second antibodies (1/5000). Bands on the blot were visualized with a chemiluminescent detection kit (P90720, Millipore, MA, USA). Publicly available ImageJ software was used to quantify the fluorescence density of the bands.

### Terminal Deoxynucleotidyl Transferase dUTP Nick End Labeling Analysis

To observe apoptotic cells, a TUNEL detection kit (#12156792910) purchased from Roche (Indianapolis, IN, USA) was used to show the fluorescence of stained apoptotic cells. The detailed protocol used for this experiment was described elsewhere. Images were obtained from a Zeiss immunoﬂuorescence microscope (Zeiss, Germany).

### Measurement of the Mitochondrial and Cytosolic Ca^2+^ Concentration

U251 and U87 cells grown on Petri dishes were individually administered 2 μM Rhod-2/AM or Fluo-2, incubated at 37°C for 30 min in Hank’s balanced salt solution (HBSS, Gibco BRL, San Diego, CA) and then washed 3 times with PBS. After another 20-min incubation in HBSS at 37°C in a humidified incubator in 5% CO_2_, images were recorded. After 3 min of baseline recording, CaCl_2_ was added to a final concentration of 2 mM, and a constant image was recorded for 7 min with an inverted microscope at 488 and 561 nm excitation using a 40× objective (Zeiss, Germany).

### Measurement of the MMP

U251 and U87 cells were preloaded on slides overnight. After treatment, 5,5′,6,6′-tetrachloro-1,1′,3,3′-tetraethylimidacarbocyanineiodide (JC-1, C2006) from an MMP assay kit (Beyotime Co., Shanghai) was used to stain the cells for 30 min. Images were obtained with a Zeiss immunoﬂuorescence microscope at 488 and 561 nm excitation using a 20× objective (Zeiss, Germany).

### Staining to Observe Mitochondrial Morphology

MitoTracker Red (M7512) purchased from Invitrogen (California, USA) was used to stain mitochondria to observe their morphology. First, either U251 or U87 cells were plated on slides overnight. After treatment, MitoTracker Red (1/10000) was added and incubated for 1 h, and the slides were then washed with PBS three times. The slides were subsequently fixed and observed under an Olympus confocal microscope (Olympus, Japan). The mitochondrial fragmentation and length were measured by Image J.

### Determination of Mitochondrial Mass and Mitochondrial DNA

To detect variation in mitochondrial mass, MitoTracker Red was used in the process. Cells were treated as described. The cells were centrifuged at 12,000 rpm for 5 min and resuspended in PBS. 100 nM of MitoTracker Red was added, and the cells were incubated for 30 min at growth condition. Last, the fluorescence intensity was measured with a FACSVerse flow cytometer.

The mtDNA copy number were measured by quantitative real-time polymerase chain reaction (qRT-PCR). The primer sequences of MT-ND1 gene was used for mtDNA amplification. The primer sequences were as follows: forward primer, 5’-ACACTAAGGTTTGGGTTTGGGTTTGGGTTTGGGTTAGTGT-3’; reverse primer, 5’-TGTTAGGTATCCCTATCCCTATCCCTATCCCTATCCCTAACA-3’. β-actin was used as a housekeeping gene control in all reaction, and the primer pair was as follows: forward primer, 5’-CACCCAGCACAATGAAGATCAAGAT-3’; reverse primer, 5’- CCAGTTTTTAAATCCTGAGTCAAGC-3’.

### Tumor Heterograft Study

This experiment was approved by the Institutional Animal Care and Use Committee of Jinling Hospital and Nanjing Medical University (Nanjing, China). First, a stable CTSS knockdown U87 cell line was established. Then, approximately 1 × 10^7^ U87 cells (from the shCtrl or shCTSS group) were transplanted subcutaneously into the right ﬂanks of male BALB/c nude mice (4 to 6 weeks old). The tumor volume (V) was calculated with the following formula: V (mm^3^) = (major axis) × (minor axis)^2^ × 0.5236. The tumor volume was recorded every 3 days. Finally, mice were sacrificed, and the tumors were extracted, fixed, and embedded in paraformaldehyde for immunohistochemical (IHC) analysis and immunofluorescence (IF) staining.

### IF Staining of Cells and Tissue

Cells were seeded onto a circular microscope cover glass overnight and subsequently treated. Cells on the slides were stained for cytochrome c (1/200) or LC3 (1/200). Mouse tumor tissues were stained for CD31 (1/200). Briefly, slides were fixed with paraformaldehyde for 15 min and then treated with 0.3% Triton X-100 for 10 min. After the slides were blocked with fetal bovine serum for 30 min, specific primary antibodies and matched secondary antibodies were used to sequentially stain the slides. Finally, nuclei were stained with DAPI (Sigma-Aldrich, D9542, 1/2000) for 10 min. All the slides and tumor sections were observed with a Zeiss immunoﬂuorescence microscope (Zeiss, Germany).

### IHC Analysis

Tissue sections (4–6 µm) were obtained from ectopic heterograft tumor samples. The paraffin-embedded tissue sections were deparaffinized, treated with 3% H_2_O_2_, and incubated with primary antibody, followed by incubation with HRP-labeled secondary antibody. The visual signal was developed with 3,3′-diaminobenzidine, and the tissue sections were then observed and photographed with a Zeiss immunoﬂuorescence microscope (Zeiss, Germany).

### Statistical Analysis

SPSS version 22.0 (IBM Corporation, Armonk, NY, USA) software was employed to analyze all the data. Data are expressed as the mean ± SEM and were evaluated by Student’s *t*-test and ANOVA for multiple comparisons. *p*<0.05 was used as a gauge of statistical significance.

## Results

### CTSS Is Upregulated in GB, and the Deletion of CTSS Reduces GBC Viability and Increases TMZ Sensitivity

**Figure 1:**
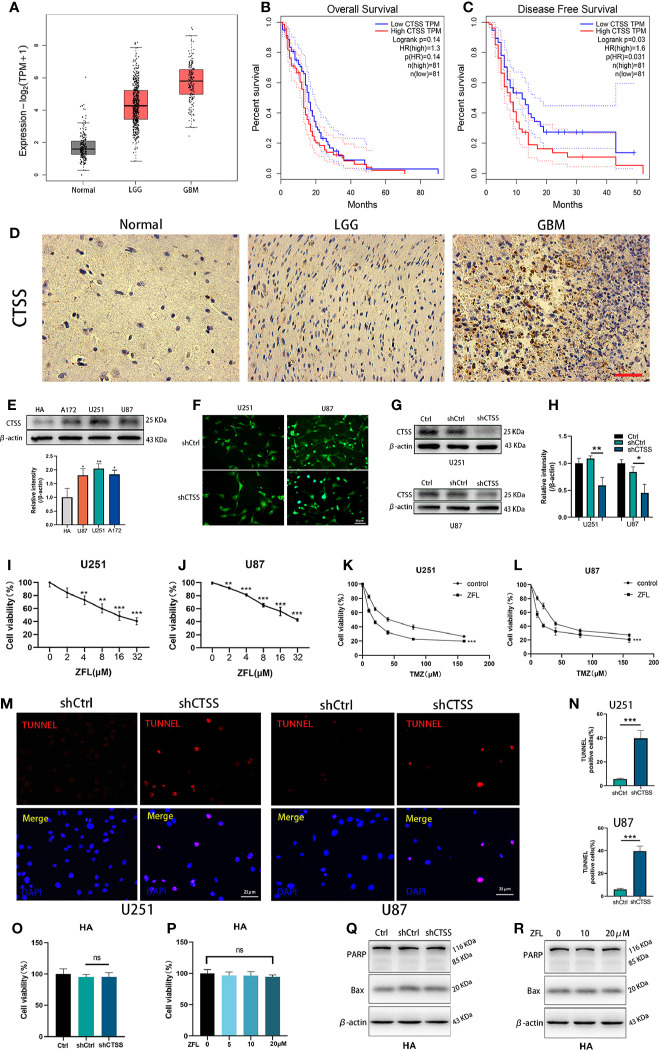
CTSS is upregulated in glioblastoma, and the deletion of CTSS reduces GBC viability and increases TMZ sensitivity. **(A)** Boxed plots from the GEPIA database were used to compare the expression of *CTSS* in mormal tissues, low grade glioma (LGG) and GBM. p _LGG/Normal_<0.05, p _GBM/Normal_<0.05. **(B, C)** OS and DFS were compared between the low-*CTSS* group and high-*CTSS* group. **(D)** The expression of CTSS of human tissues was assessed by immunohistochemical staining. **(E)** The expression of CTSS in HAs and the U87, U251, and A172 cell lines was detected by western blotting. The level of CTSS was clearly increased in the A172, U251, and U87 cell lines compared with that in human astrocytes (HAs). **(F–H)** Cells were transfected with shCtrl or shCTSS lentivirus labeled with GFP. Scale bar, 50 μm. Western blotting showed that CTSS was obviously decreased in the shCTSS groups. **(I, J)** The U87 and U251 cell lines were treated with ZFL at the indicated concentrations (0, 2, 4, 8, 16, 32 μM) for 48 h. The CCK-8 assay showed that the viability of U251 and U87 cells was decreased in a dose-dependent manner. **(K, L)** U87 and U251 cells were transfected with shRNA. The shCtrl groups and shCTSS groups were separately treated with the indicated concentrations of TMZ (0, 10, 20, 40, 80, 160 μM) for 8 h, and cell viability was then detected by CCK-8 assay. **(M, N)** Cell death was detected by TUNEL assays after cells were transfected with shRNA for 48 h. Scale bar, 25 μm. Human normal astrocyte (HA) cell line was transfected with CTSS-KD virus or scramble virus. **(O, P)** Cell viability was tested by CCK-8 assay. **(Q, R)** Apoptosis-related proteins were tested by Western blot. The results showed that no significant change was observed between the shCtrl groups and the shCTSS groups. Data are represented as the means ± SEMs of three independent experiments. *p < 0.05, **p < 0.01, ***p < 0.001 versus the control group.

CTSS mRNA expression was significantly higher in low grade glioma (LGG) and GB samples than in normal samples according to TGCA data (**Figure 1A**). Then, we estimated the prognostic value of CTSS in GB by the Kaplan-Meier survival analysis plotter. Though OS of high-*CTSS* group was not significantly decreased when compared with low-*CTSS* group (p=0.14), high expression of *CTSS* mRNA correlated with decreased DFS (p=0.03) (**Figures 1B, C**). Immunohistochemical staining with CTSS confirmed that CTSS was highly expressed in GB tissue (**Figure 1D**). Then, we detected the expression of CTSS in GBCs by western blotting. The expression of CTSS was significantly upregulated in the human GBC lines (U87, U251, and A117) compared with normal HAs (**Figure 1E**). This finding was consistent with those of our previous study. Subsequently, to verify the functional role of CTSS in GBC phenotypes, lentivirus targeting CTSS was developed. Western blotting indicated a significant decrease in CTSS in GB knockdown cells (**Figures 1F–H**). After the inhibition of CTSS with ZFL at different concentrations of, CCK8 assays were used to analyze the viability of GBCs. We found that cell viability was decreased in a dose-dependent manner (**Figures 1I, J**). Moreover, cell viability assays showed that CTSS knockdown GBCs were susceptible to lower concentrations of TMZ than the control group, suggesting that the knockdown of CTSS increased the sensitivity of the GBC lines to TMZ (**Figures 1K, L**). Furthermore, TUNEL staining was employed, and more TUNEL-positive cells were found in the shCTSS groups than in the control groups, suggesting that targeting CTSS has a killing effect on GBCs (**Figures 1M, N**). We also examined the effect of CTSS inhibition on human astrocyte line (HA). Viability tests and apoptosis-related proteins detection showed no significant change between control groups and shCTSS groups (**Figures 1O–R**), indicating CTSS inhibition could not affect HA viability.

### Inhibition of CTSS Induces Ca^2+^ Redistribution and MCU Complex in GBC Lines

**Figure 2:**
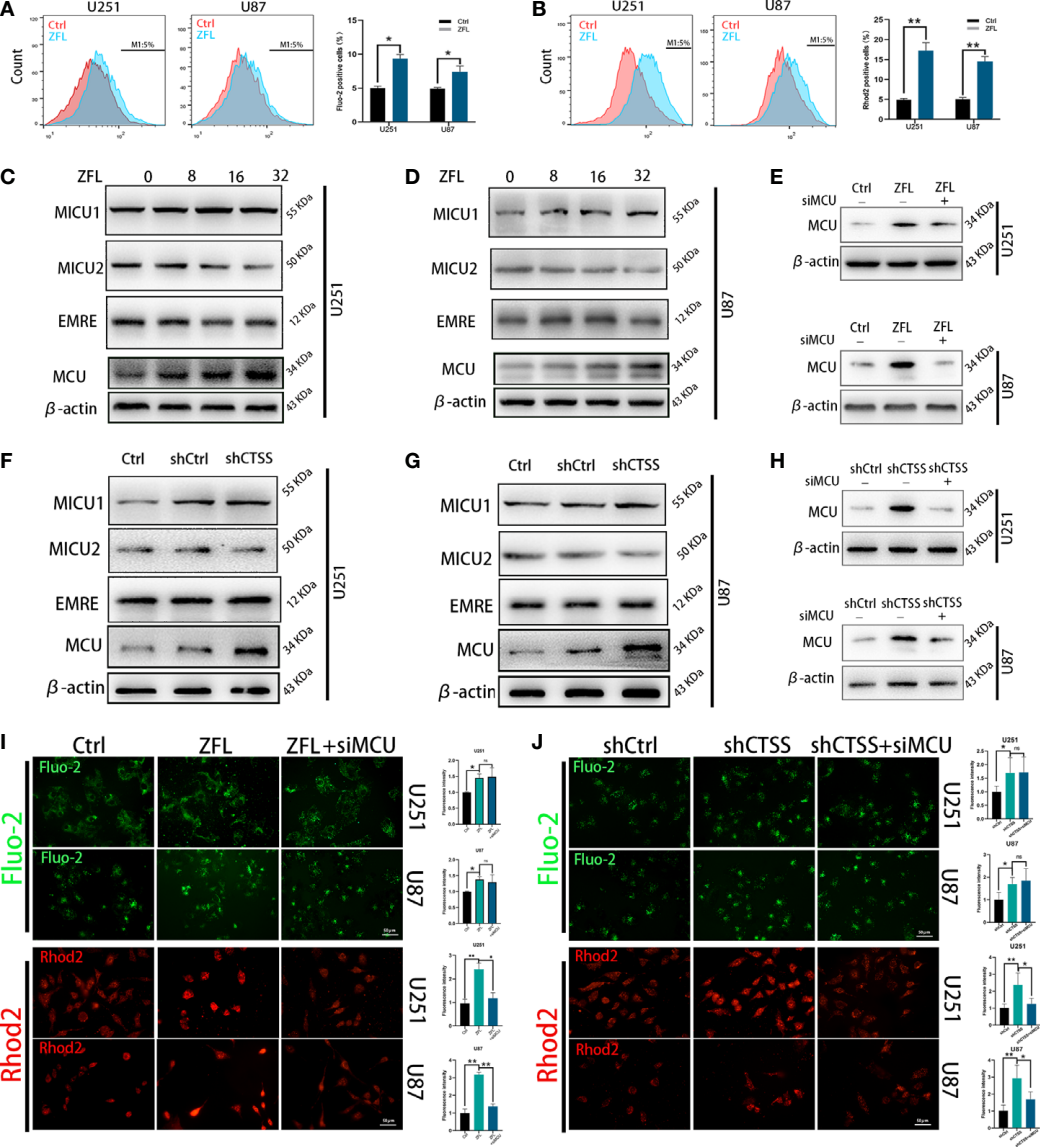
Inhibition of cathepsin S redistributes Ca^2+^ levels and alters MCU complex expression in glioblastoma cell lines. Cells were treated with 20 μM ZFL for 48 h. In the ZFL+siMCU groups, siRNA targeting MCU was added. **(A, B)** Cells were stained with Fluo-2 or Rhod-2 as previous described by flow cytometry. Then intracellular Ca2+ **(A)** and mitochondrial Ca2+ **(B)** were measured by flow cytometry. **(C, D, F, G)** The expression of MCU, MICU1, MICU2 and EMRE among the control groups, shCtrl groups, and shCTSS groups and groups treated with different concentrations of ZFL was measured by western blotting. β-Actin was used as an internal control. **(E, H)** Protein levels of MCU after adding siMCU were accessed by western blotting. **(I, J)** Then, intracellular and mitochondrial Ca^2+^ was imaged by the fluorescence staining of Fluo-2 and Rhod2. Scale bar, 50 μm. Data are represented as the means ± SEMs of three independent experiments. *p < 0.05, **p < 0.01 versus the control group.

To explore the mechanisms by which CTSS regulates ROS generation and because the Ca^2+^ distribution was found to be related to mitochondrial energy metabolism and ROS generation, we evaluated cytoplasmic and mitochondrial Ca^2+^ levels. By staining Fluo-2 or Rhod-2 and then detected by flow cytometry, we observed that the cytoplasmic Ca^2+^ level was moderately increased (**Figure 2A**), but the mitochondrial Ca^2+^ level of the ZFL groups was obviously elevated (**Figure 2B**), indicating that the mitochondria in the ZFL groups had an enhanced ability to take up Ca^2+^.

To determine whether MCU plays an underlying role in this effect, we detected the expression of MCU and other components of MCU complex by western blotting. As shown in **Figures 2C–D, F–G**, the protein levels of MCU were significantly higher in the ZFL-treated groups and the shCTSS groups than the control groups, demonstrating that MCU-mediated Ca^2+^ uptake could be crucial to the effects of CTSS knockdown. We also observed no obvious change of EMRE level, and slight increase of MICU1 level and mild decrease of MICU2 level. MCU expression is related to mitochondrial Ca^2+^ elevation and MICU1 expression favors Ca^2+^ uptake by MCU channel while MICU2 could stop Ca^2+^ entering mitochondria. Therefore, MCU complex change may contribute to CTSS-mediated mitochondrial Ca^2+^ overload. To confirm this finding, we used an MCU siRNA to decrease MCU protein expression (**Figures 2E, H**). We found that MCU knockdown significantly decreased mitochondrial Ca^2+^ levels in the ZFL-treated groups and the shCTSS groups compared with the control groups (**Figures 2I, J**). We also observed a slight elevation in cytoplasmic Ca^2+^ in the ZFL+miMCU groups compared with the ZFL-treated groups, but no statistical difference was recorded (**Figures 2I, J**).

### Inhibition of CTSS Influences Mitochondrial Biological Properties Through MCU

**Figure 3:**
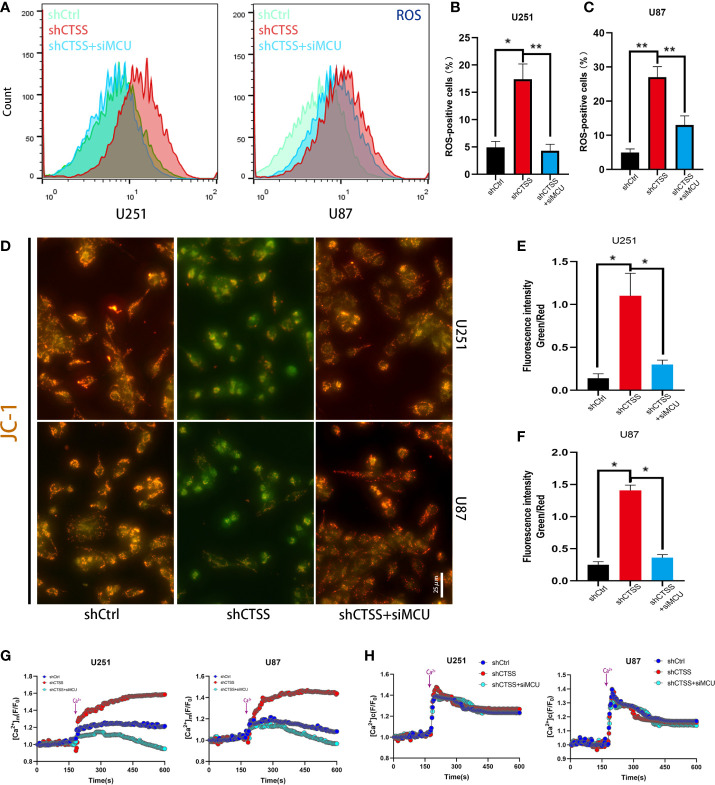
Inhibition of CTSS influences mitochondrial biological properties through MCU. **(A–C)** Cells in which CTSS was knocked down were established. ROS levels were then measured by flow cytometry. **(D, F)** The mitochondrial membrane potential was determined by JC-1 staining. Scale bar, 25 μm. **(G, H)** Mitochondrial and cytosolic Ca^2+^ was measured by the fluorescence staining of Rhod-2 and Fluo-2 respectively; dynamic changes in mitochondrial and cytosolic Ca^2+^ were recorded as illustrated above. Data are represented as the means ± SEMs of three independent experiments. *p < 0.05, **p < 0.01 versus the control group.

We further confirmed that ROS levels were elevated after the knockdown of CTSS in the U251 and U87 cell lines (**Figures 3A–C**). However, ROS levels were significantly decreased in shCTSS+siMCU groups when compared with shCTSS groups (**Figures 3A–C**). To investigate whether mitochondrial properties were influenced by the inhibition of CTSS in GBC lines, we first detected the MMP by JC-1 staining. With this technique, cells with a damaged MMP are stained green, while cells with a normal MMP are stained red. As shown in **Figures 3D–F**, pictures of the shCTSS cell lines contained an increased green stained intensity and a decreased red stained intensity compared with those in pictures of the control or shCTSS+siMCU groups, indicating the loss of MMP in the shCTSS groups and MMP recovery in the ZFL+siMCU groups.

Then, we detected the capability of mitochondria to take up Ca^2+^. First, we recorded baseline fluorescence of Rhod-2AM for 3 min, following which Ca^2+^ at a final concentration of 2 mM was added, and the fluorescence signal was recorded for another 7 min. The results showed that the fluorescence increased rapidly after Ca^2+^ was added to the control and shCTSS groups (**Figure 3G**). However, an increased amplitude was observed in the shCTSS groups, which indicated a Ca^2+^ outburst that may contribute to mitochondrial stress. In contrast, the fluorescence intensity measured in the shCTSS+siMCU groups increased less rapidly and reached a lower amplitude (**Figure 3G**). Meanwhile, dynamic cytosolic detection Ca^2+^ indicated that CTSS knockdown or MCU inhibition has little effect on the dynamic change of cytosolic calcium (**Figure 3H**). Similar to mitochondrial detection, cytosolic Ca^2+^ concentration was recorded by staining of Fluo-2AM. Baseline cytosolic Ca^2+^ concentration was standardized. Baseline fluorescence of Flou-2AM was recorded for 3 min before 2mM Ca^2+^ was added. However, no obvious difference was recorded between the groups (**Figure 3H**), indicating that cytosolic Ca^2+^ wave was not obviously influenced.

**Figure 4:**
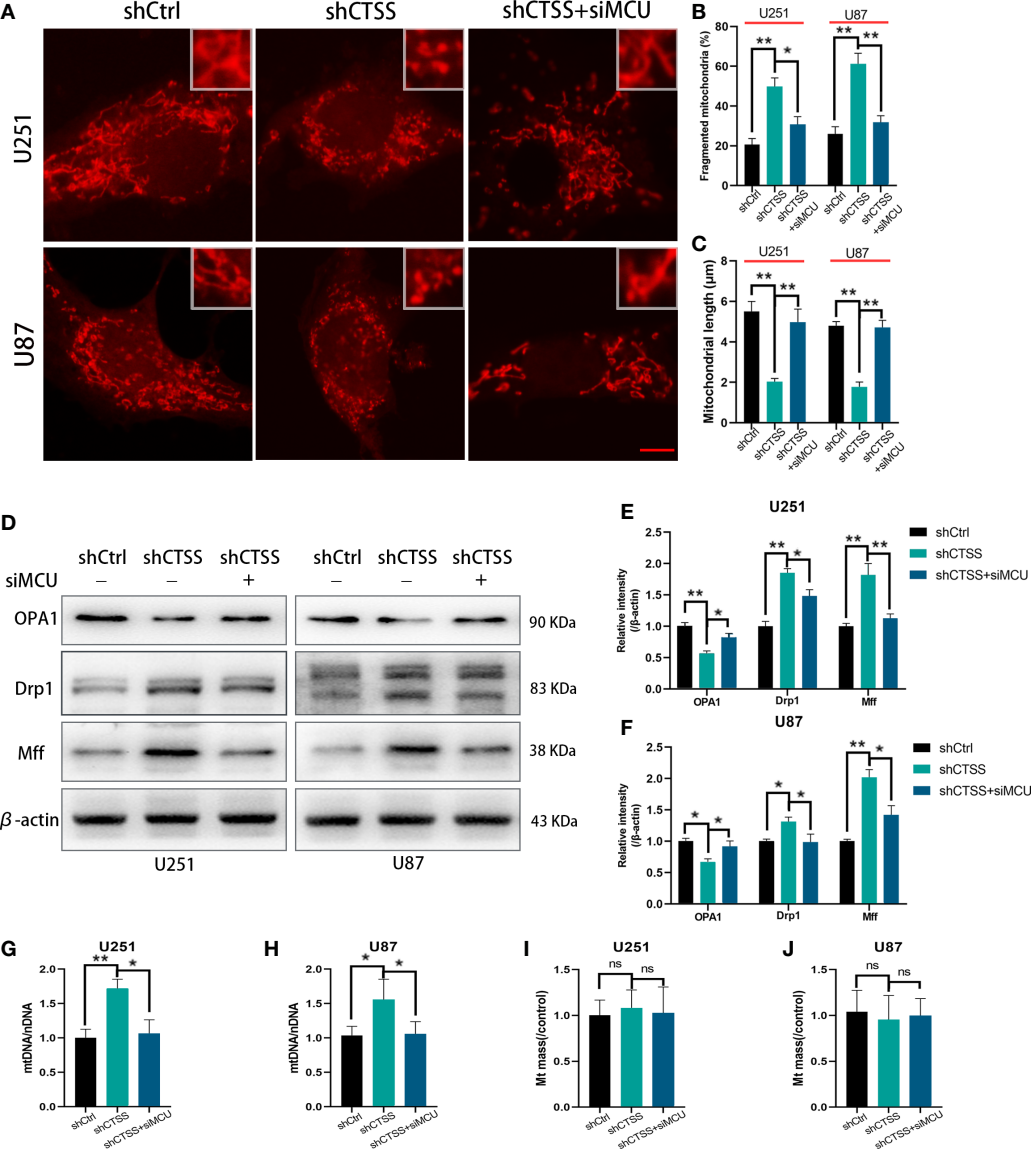
Inhibition of CTSS influences mitochondrial morphology change. U251 and U87 cells were treated with lentivirus targeting CTSS, with or without adding siMCU. **(A–C)** Mitochondrial morphology was stained by MitoTracker Red and then pictured by confocal microscopy. The mitochondrial fragmentation and length were measured by Image J. Scale bar, 10 μm. **(D–F)** Western blot was applied to detect mitochondrial fission and fusion associated protein Mff, Drp1 and OPA1. **(G–J)** mtDNA and mitochondrial mass were measured as described. Data are represented as the means ± SEMs of three independent experiments. *p < 0.05, **p < 0.01 versus the control group.

Mitochondrial morphology change is another aspect of mitochondrial damage. The percentage of fragmented mitochondria was elevated and the mitochondrial length was decreased in the shCTSS groups (**Figures 4A–C**). These effects could be reversed by silencing MCU (**Figures 4A–C**). However, whether mitochondrial fission was related to CTSS knockdown-induced mitochondrial damage and how MCU took part in this process need further studies. Therefore, we detected mitochondrial fission and fusion related protein, Mff, Drp1, and OPA1. The results indicated that mitochondrial fission related proteins Mff and Drp1 were elevated in shCTSS group comparing with shCtrl group and dropped in the shCTSS+siMCU group (**Figures 4D–F**). Meanwhile, mitochondrial fusion related protein OPA1 showed an opposite change (**Figures 4D–F**). Further, we detected mitochondrial DNA. As shown in **Figures 4G, H**, mitochondrial DNA was elevated in the shCTSS groups compared with the shCtrl groups and decreased when compared with shCTSS+siMCU groups. However, mitochondrial mass between three groups was not obviously changed (**Figures 4I, J**).

These results suggested that knockdown of CTSS impedes mitochondrial metabolism through MCU.

### CTSS Deletion Induces Mitochondrial Apoptosis Through MCU

**Figure 5:**
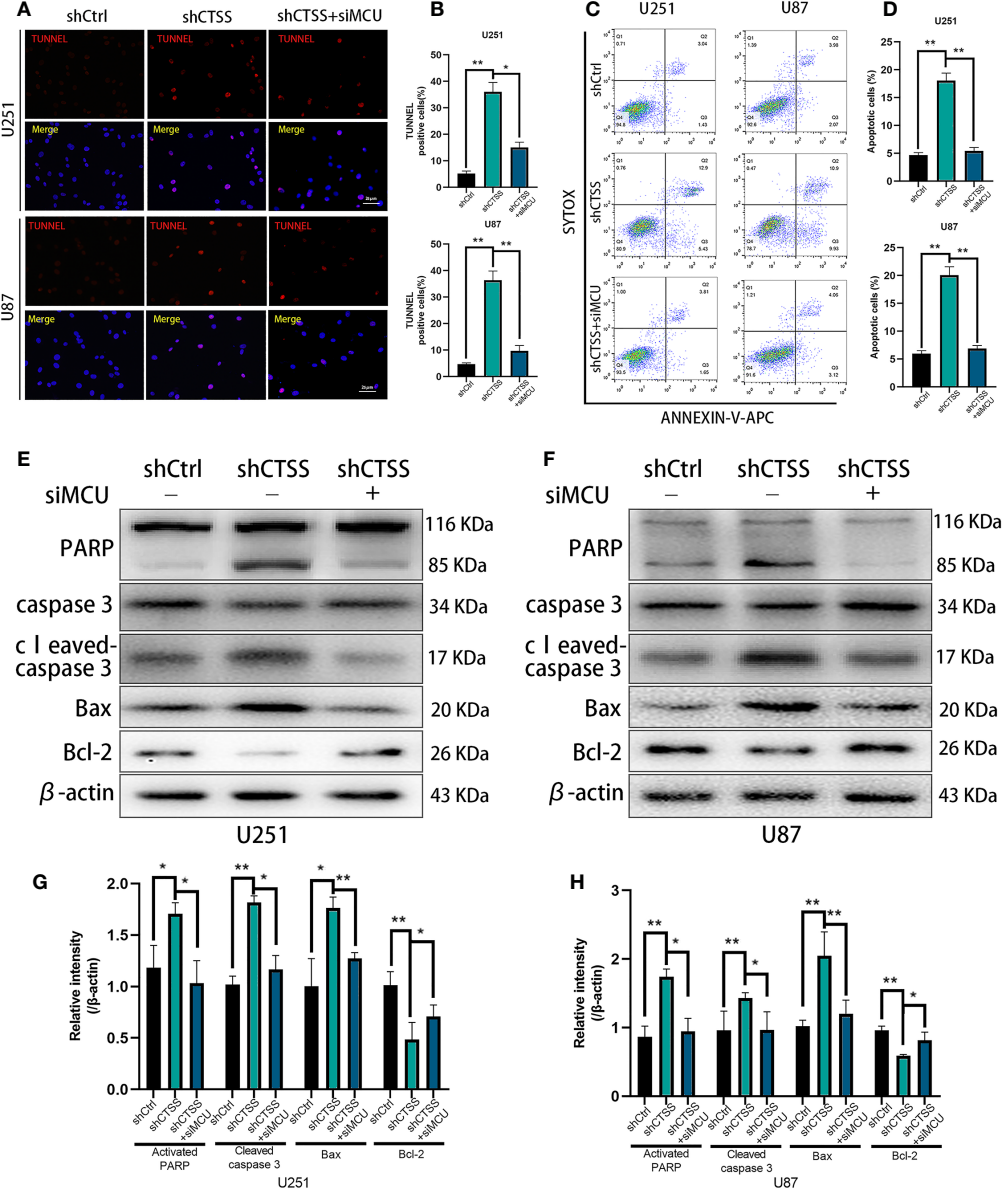
CTSS deletion induces mitochondrial apoptosis through MCU. Cells were treated as described. **(A–D)** Apoptosis in different groups was detected by TUNEL staining and flow cytometry. The proportions of apoptotic cells in the shCTSS groups were significantly elevated, and the proportions of apoptotic cells were decreased in the shCTSS+siMCU groups. **(E–H)** The expression of caspase-3, cleaved caspase-3, PARP, Bcl-2, and Bax was detected by western blotting. β-Actin was used as an internal control. Data are represented as the means ± SEMs of three independent experiments. *p < 0.05, **p < 0.01 versus the control group.

We previously observed that the inhibition of CTSS in GBC lines could induce apoptosis. Here, we explored whether MCU is involved in this process. First, through TUNEL staining and flow cytometry, we observed an increased proportion of apoptotic cells in the shCTSS groups compared with the control groups. However, the proportion of apoptotic cells was significantly decreased in the shCTSS+siMCU groups (**Figures 5A–D**).

**Figure 6:**
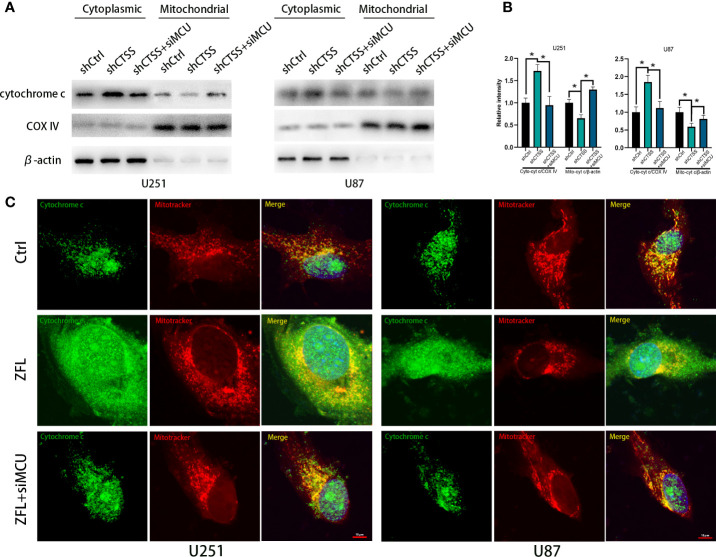
CTSS deletion induces cytochrome c release from the mitochondria. Cells were treated as above. **(A, B)** The levels of cytochrome C in the mitochondria and cytoplasm were measured by western blotting. **(C)** Cells were first dyed with MitoTracker Red. After washing with PBS three times, the cells on slides were fixed with paraformaldehyde. The localization of cytochrome c was subsequently measured by immunofluorescence staining. As shown in this picture, mitochondria in the ZFL-treated groups tended to appear as particles. Moreover, the cytochrome c fluorescence was more dispersed in the ZFL-treated groups compared with the control groups and ZFL+siMCU groups. Scale bar, 10 μm. Data are represented as the means ± SEMs of three independent experiments. *p < 0.05 versus the control group.

Then, we detected the expression of apoptosis-related proteins by western blotting. We observed the decreased expression of Bcl-2 accompanied by an increased level of Bax in the shCTSS groups (**Figures 5E–H**). Since Bcl-2 is one of the most important and multifunctional antiapoptotic proteins, the loss of Bcl-2 could affect the ability of cells to adapt to stress and lose control of proapoptotic proteins such as Bax and Bad (**Figures 5E–H**), which could further activate downstream members of apoptosis signaling pathways, such as cytochrome c. Therefore, we examined whether cytochrome c was released from the mitochondria (**Figures 6A, B**). Through fluorescence staining for cytochrome c, the addition of ZFL to U87 and U251 cell lines was shown to prompt cytochrome c fluorescence to become more dispersed compared with that in the control groups (**Figure 6C**). Then, through western blotting, we found that the expression of cleaved caspase 3 and PARP was elevated (**Figures 5E–H**). All of these results indicated that caspase-dependent apoptosis was activated in the shCTSS groups. However, following siMCU treatment, we found elevated levels of Bcl-2, decreased levels of Bax, greater overlap between cytochrome c fluorescence and mitochondria, and downregulation of cleaved caspase 3 and PARP (**Figures 5E–H**, **6A–C**). These results suggested that blockade of MCU could rescue the CTSS-induced loss of apoptosis.

### CTSS Deletion Induces Autophagosome Accumulation, Which Is Partly Relieved by the Blockade of MCU

**Figure 7:**
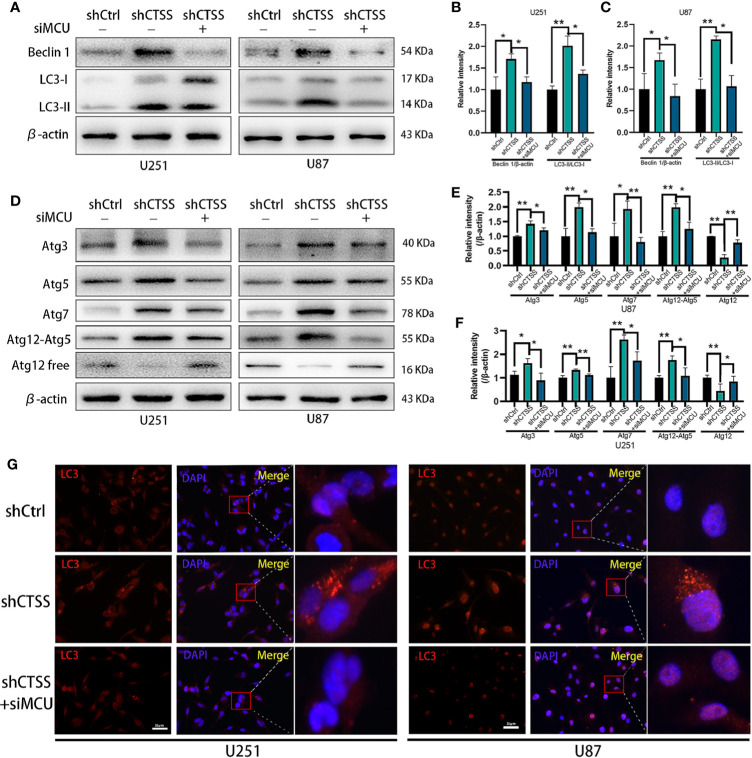
CTSS deletion induces elevation of autophagy-related proteins, which is partly relieved by the blockade of MCU. Cells were arranged into three groups. Each group was treated as described. **(A–F)** The expression of Beclin 1, LC3, Atg3, Atg5, Atg7, and Atg12 was measured by western blotting. β-Actin was used as an internal control. The levels of autophagy-related proteins were changed in the shCTSS groups, and these changes were partly reversed by silencing MCU. **(G)** Autophagosomes were detected by immunofluorescence staining form LC3. More LC3-positive particles were observed in the shCTSS groups than in the shCtrl groups and the shCTSS+siMCU groups. Scale bar, 25 μm. Data are represented as the means ± SEMs of three independent experiments. *p < 0.05, **p < 0.01 versus the control group.

Our previous studies found that autophagy is essential for inhibiting CTSS-induced apoptosis. Here, we wondered whether MCU is associated with this process. As shown in **Figures 7A–C**, knockdown of CTSS could induce LC3 conversion, but blockade of MCU inhibited the conversion of LC3 to LC3-II. We also examined the change in Beclin 1 expression and found that the siMCU could reverse the elevated expression of Beclin 1 induced by the knockdown of CTSS (**Figures 7A–C**). Moreover, the other autophagy-related proteins Atg3, Atg5, Atg7, and Atg12 were influenced accordingly by the blockade of MCU (**Figures 7D–F**). We also detected the formation of autosomes by immunofluorescence staining for LC3II. As shown in **Figure 7G**, a higher density of LC3II-positive particles was observed in the shCTSS groups, while these particles were hardly observed in the control and shCTSS+siMCU groups.

**Figure 8:**
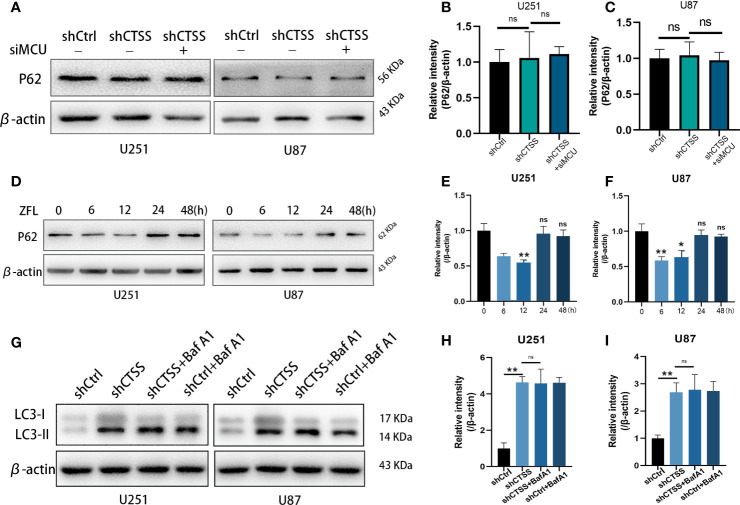
CTSS deletion induces blockade of autophagic flux. **(A–C)** Cells were treated as described. The expression levels of p62 were measured by western blotting. However, no significant change in p62 was recorded. **(D–F)** p62 protein levels after GB cells treated with ZFL for different hours were detected by Western blot. The results showed that the level of p62 decreased within 12 h but started to recover after 12 h. **(G–I)** Western blot was used to examine the autophagic flux by LC3 turnover assay. We observed that LC3-II accumulation in shCTSS groups, and BafA1 could not augment LC3-II accumulation in shCTSS+BafA1 groups compared with shCTSS groups. Data are represented as the means ± SEMs of three independent experiments. *p < 0.05, **p < 0.01 versus the control group.

We then evaluated the expression of p62, which indicates the process of autophagosome and lysosome fusion. We found that the expression of p62 was not significantly decreased in the shCTSS groups (**Figures 8A–C**), indicating that the knockdown of CTSS could impair lysosome function and decrease the rate of autophagosome degradation and autophagic flux. However, our previous study showed that GB cell lines treated with ZFL for 8 h decreased the level of p62 protein, indicating enhanced autophagic flux activity. Since shCTSS groups were treated with lentivirus against targeted gene for more than 2 days to ensure stable knockdown of CTSS, we deduced that the duration of CTSS inhibition contributed to different stage of autophagic activity. Therefore, we detected p62 protein level after GB cells treated with ZFL for different hours. Western blot results showed that the level of p62 decreased within 12 h but started to recover after 12 h (**Figures 8D, E**), indicating long-term CTSS inhibition probably cause blockade of autophagic flux. To detect whether or not autophagic flux was shut in this process, we then further examine the autophagic flux by LC3 turnover assay. Treatment with BafA1, an inhibitor that blocked autophagy at the late stages, could result in an increase in LC3-II accumulation (**Figures 8G, H** **)**. We observed that LC3-II accumulation in shCTSS groups, and BafA1 could not augment LC3-II accumulation in shCTSS+BafA1 groups compared with shCTSS groups (**Figures 8G, H** **)**. Collectively, these data illustrated the autophagic flux in glioblastoma could be blocked in long-term CTSS inhibition.

### Cytosolic Ca^2+^ Elevation Is Critical for CTSS-Inhibition Induced MCU Expression

**Figure 9:**
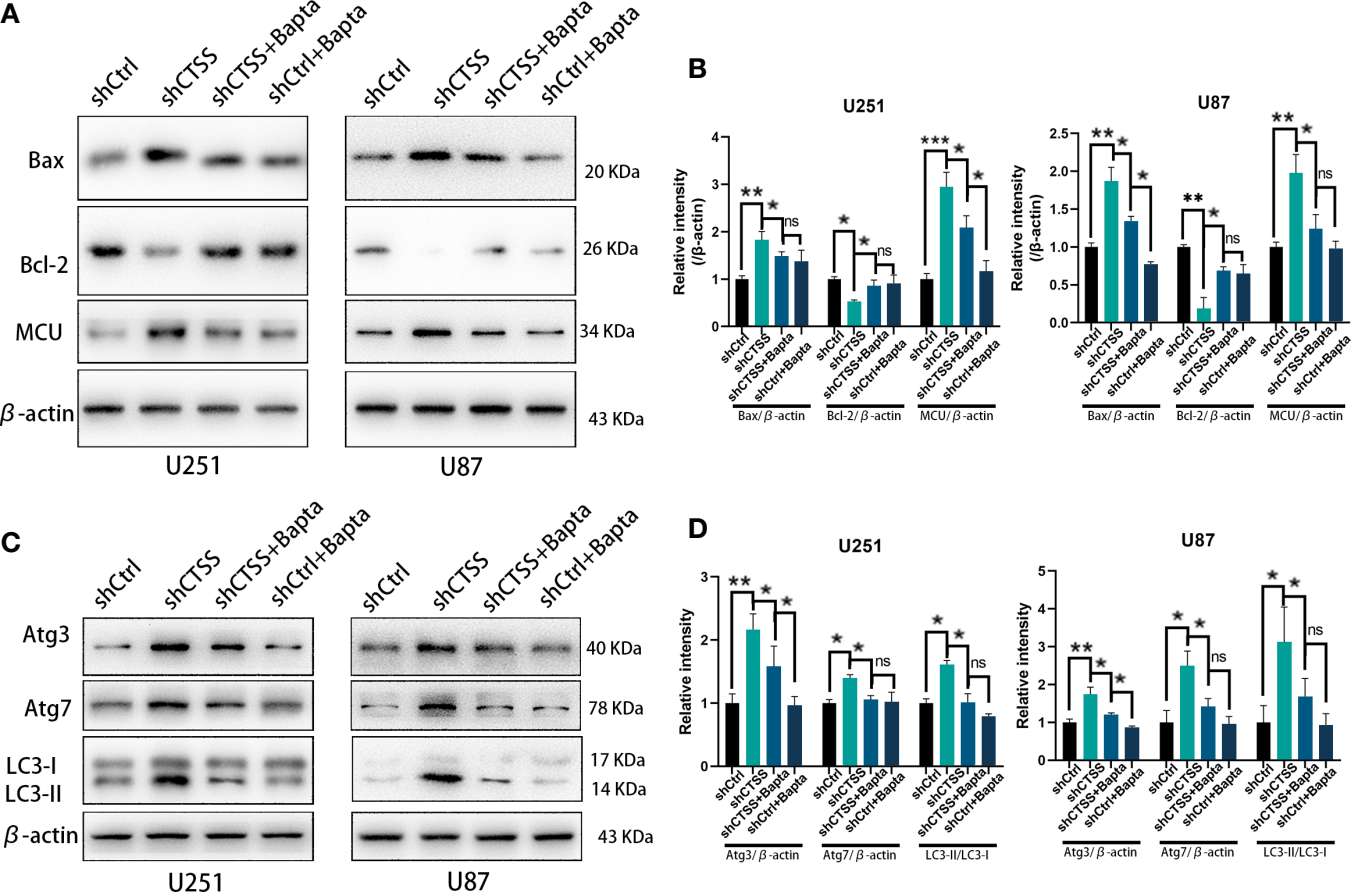
Cytosolic Ca^2+^ elevation is critical for CTSS-inhibition induced MCU expression. Cells with or without CTSS knockdown was established as described. 5 µM BAPT-AM, a Ca^2+^ chelator, was employed to inhibit the up-regulation of intracellular Ca^2+^ levels. **(A, B)** MCU, Bcl-2 and Bax were measured by Western blot. MCU protein levels were elevated in the shCTSS groups, and adding BAPTA-AM could partly decrease MCU levels. Meanwhile, BAPTA-AM could alleviate pro-apoptosis protein Bax protein levels and recuperate anti-apoptosis protein Bcl-2 protein loss induced by CTSS inhibition. **(C, D)** Autophagic proteins were also detected by Western blot under the condition of BAPTA-AM adding. Atg3 and Atg7 and LC3-II were recorded upregulation in shCTSS groups but decreased as long as BAPTA-AM was used. Data are represented as the means ± SEMs of three independent experiments. *p < 0.05, **p < 0.01 versus the control group.

Since cytosolic Ca^2+^ elevation was observed in our study, further we investigate the role of intracellular Ca^2+^ change on MCU expression and autophagic activity after CTSS deletion. BAPTA-AM, a Ca^2+^ chelator, was employed to inhibit the up-regulation of intracellular Ca^2+^ levels. As shown in the **Figures 9A, B**, MCU protein levels were elevated in the shCTSS groups, adding BAPTA-AM could partly decrease MCU levels. Meanwhile, we also detected apoptosis-related proteins and found that BAPTA-AM could alleviate pro-apoptosis protein Bax protein levels and recuperate anti-apoptosis protein Bcl-2 protein loss induced by CTSS inhibition. These results demonstrated that CTSS deletion induced cytosolic Ca^2+^ elevation was critical for MCU expression and subsequent apoptosis.

Additionally, autophagic proteins were also detected under the condition of BAPTA-AM adding. Atg3 and Atg7 and LC3-II were recorded upregulation in shCTSS groups but partly decreased as long as BAPTA-AM was used (**Figures 9C, D**). This result indicated that CTSS-inhibition induced intracellular elevation may be responsible for autophagy change and primary motive for morbid state of cells.

### Knockdown of CTSS Inhibits Tumor Growth *In Vivo*

**Figure 10:**
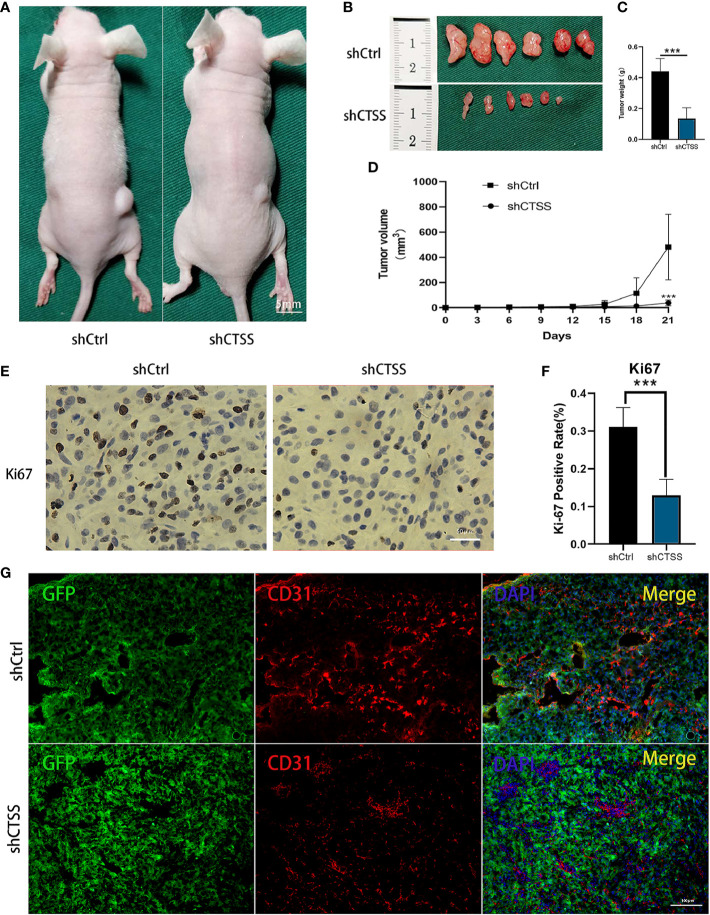
Knockdown of CTSS inhibits tumor growth *in vivo*. **(A–D)** U87 cells with stable knockdown of CTSS were established and then used to inoculate nude mouse. The growth of tumors in the shCtrl group and shCTSS group was calculated as described above. The growth curves of tumors in the two groups were generated (n=6). **(E, F)** Immunohistochemical staining for Ki-67 was used to detect the tumor proliferation state. Scale bar, 50 μm. Ki-67-positive cells in six independent fields were quantified. **(G)** Tumor vascularity was visualized by immunofluorescence staining for CD31. Scale bar, 100 μm. Data are represented as the means ± SEMs of independent experiments. ***p < 0.001 versus the vehicle group.

We further determined whether knockdown of CTSS could suppress the growth of GB in nude mice heterografted with U87 shCtrl or shCTSS cells. Tumor volume was measured every 3 days, and tumor growth curves were generated for the different groups. As shown in **Figures 10A–D**, compared with the control group, the tumor volume was significantly smaller in the shCTSS groups. Then, the expression of Ki-67, a biomarker of cancer proliferation, was detected by immunohistochemical staining of samples from the xenografted tumors. The shCTSS groups showed fewer Ki-67-positive cells than the control groups (**Figures 10E, F**). Moreover, the tumor vascularity was determined by IF staining for CD31. As shown in **Figures 10G**, the vessel diameters in tumor samples from the shCTSS groups were much smaller than those in the control groups, which suggested that the shCTSS cells had a slower growth rate.

## Discussion

This study mainly demonstrated the potential role of targeting CTSS for GB treatment. We found that inhibition of CTSS exert anti-tumor effects both *in vitro* and *in vivo*. Obvious change of MCU complex was observed in our study: an elevated protein level of MCU and increased mitochondrial Ca^2+^ levels after CTSS inhibition. Further studies found that the MMP was decreased, ROS were generated, and apoptosis-related proteins were released in the CTSS-inhibited groups; and blockade of MCU could reverse these detrimental effects induced by CTSS knockdown. As an important protease from the lysosome, knockdown of CTSS could affect autophagy in GBC lines (8). Knockdown of MCU could partly decrease CTSS-inhibition induced autophagy-related proteins elevation. Accidently, we found that autophagy-related proteins were highly expressed, but the expression of p62 was not significantly changed in our present study, which indicated impaired lysosomal function and obstructed autophagic flux. Autophagic flux test confirmed that knockdown of CTSS in glioblastoma contribute to blockade of autophagic flux. We also found that chelation of cytosolic Ca^2+^ by Bapt-AM could partly decrease MCU level induced by CTSS inhibition.

High expression of CTSS in different cancers, including GB, is a dangerous factor that can contribute to tumor proliferation, migration and invasion (7, 27–30). In addition to inhibiting tumor invasion and angiogenesis (31), we found that the inhibition of CTSS in GBC lines induced autophagy and apoptosis and that these effects were strongly related to ROS-mediated PI3K/AKT/mTOR signaling pathways (8). These effects were also found in other human cancers (32, 33). In this study, we found that the expression of autophagy-related proteins was increased after the knockdown of CTSS in GBC lines, but p62 protein levels were not significantly changed, which illustrated that knockdown of CTSS may contribute to the blockade of autophagic flux. Pan L et al. found that CTSS was essential for functional lysosomes in macrophages and that knockout of CTSS could induce the abnormal accumulation of autophagosomes (34). Kamlesh Pawar et al. also found that in the context of CTSS knockdown in macrophages, the LC3B-II level was clearly increased, while the level of p62 was not decreased, which suggested the blockade of autophagic flux and impaired phagocytosis. However, we also noticed that decreased p62 levels were reported in other studies and our previous study (8, 32, 33). Most of these studies, the usage of CTSS inhibitors last less than 12 h. In this study, we conducted our experiments on stable CTSS knockdown cell lines or cells treated with ZFL for 48 h. We hypothesized that short time inhibition of CTSS could increase autophagy but induce autophagic flux blockade when CTSS inhibition endures. To prove this hypothesis, we detected p62 levels at different time point after adding ZFL and found that p62 levels decreased time-dependently within 12 h but increased after 24 h, indicating long-term CTSS inhibition by ZFL could contribute to autophagic flux blockade. Therefore, the difference between these studies probably attribute to the duration time of CTSS inhibition. Together, these data indicated that CTSS plays an important role in tumor autophagy and autophagic flux.

The blockade of autophagic flux could be detrimental to the proper functioning of mitochondria (35). Cathepsin activity has an indispensable role in the autophagy-lysosomal degradation pathway and cathepsin inhibition could contribute to accumulation of morbid mitochondria (36). Except that, previous studies also shows that knockdown of CTSS could increase the generation of ROS (8, 32), which is an important process in CTSS knockdown-related mitochondrial apoptosis. Since ROS are generated mainly by mitochondria, whether mitochondria are damaged in the context of CTSS knockdown was another focus of this study. Elevated mitochondria Ca^2+^ levels and moderately increased cytoplasmic Ca^2+^ levels were observed in the GBC lines after knockdown of CTSS, suggesting that abnormal mitochondrial Ca^2+^ elevation is the initial cause of ROS-induced apoptosis (37). Previous study found that inhibition of CTSS can cause the expression of ER stress-related proteins, as well as the increase of cytosolic Ca^2+^ in the renal cancer, lung cancer and breast cancer cell lines (9). This result shows that there is an elevation of cytosolic Ca^2+^ which is probably released from endoplasmic reticulum in the early period of inhibition of CTSS by ZFL. In order to further study whether ZFL affects IP3R [inositol 1,4, 5-trisphosphote (IP3) receptor] and RyR (ryanodine receptor) in ER, the reseachers used IP3R inhibitor 2-apb and RyR inhibitor dantrolene, and found that 2-apb and dantrolene could significantly inhibit Ca^2+^ release induced by ZFL (9). This result shows that the Ca^2+^ stored in endoplasmic reticulum can be released through IP3R and RyR in the early stage of ZFL application. This may explain why cytosolic Ca^2+^ elevated after CTSS inhibition in our study. They also investigated whether MCU participated in this process by MCU inhibitor ruthenium red (RuR). However, RuR is not an appropriate choice for blocking MCU complex in intact cells. How mitochondria react to cytosolic Ca^2+^ elevation needs further study.

Since MCU complex is a predominant Ca^2+^ uptake protein located on the inner mitochondrial membrane (24), we detected MCU complex expression and found that MCU was overexpressed in the CTSS-KD groups with MICU1 mildly elevated and MICU2 slightly decreased. MICU1/MICU2 ratio may favor mitochondria Ca^2+^ uptake (38). The increased expression of MCU was strongly related to elevated mitochondrial Ca^2+^ levels and impaired resistance to Ca^2+^ excitement, which could cause excitotoxic death (39). Block of MCU significantly decreased the inhibition of CTSS-induced mitochondrial damage and apoptosis in our study, which was consistent with previous studies (25, 40). Studies have also shown that the addition of ZFL to lung carcinoma and breast carcinoma cell lines could increase the levels of cytoplasmic Ca^2+^ and the endoplasmic reticulum (ER) stress-related proteins CHOP and ATF4, but the expression of ER stress markers was decreased after 24 h, and inhibition of CTSS failed to cause cell apoptosis (9). However, inhibition of CTSS in GBC lines caused cell apoptosis. Interestingly, mitochondrial Ca^2+^ disturbance and mitochondrial malfunction appeared much more important in apoptosis induced by CTSS knockdown in our study. This difference may be due to the different doses of ZFL used and the heterogeneity of different cell lines. Importantly, the intervention time was also different. In the previously published study, the ZFL intervention time was 8 h, which was shorter than the ZFL intervention time in our experiment (48 h). This may indicate that deletion of CTSS caused ER stress in the early stage and that this stress was transferred to mitochondria in the late stage. However, this hypothesis is just our speculation and requires clarification through further studies.

Paradoxically, enhanced MCU function and elevated mitochondrial Ca^2+^ levels usually decrease the level of autophagy (26, 40). In contrast, blockade of MCU and decreased mitochondrial Ca^2+^ levels decreased the generation of ATP and essential material supply, thus increasing autophagy (26, 41, 42). However, all these studies focused mainly on mitochondrial functions. Interestingly, in our study, we observed decreased autophagy-related protein expression in the shCTSS groups after silence of MCU. We hypothesize that knockdown of CTSS causes two kinds of effects: 1) because CTSS knockdown causes the malfunction of lysosomal function (34), autophagosomes accumulate in the cytoplasm, and damaged mitochondria cannot be eliminated in time and therefore accumulate in the cytoplasm; and 2) elevated cytoplasmic Ca^2+^ and MCU levels contribute to mitochondrial Ca^2+^ overload and cause the mitochondria to deteriorate (43), which induces mitochondrial apoptosis and exacerbates the burden of autophagy. The blockade of MCU complex partly broke this vicious circle, maintaining the balance of mitochondrial Ca^2+^ levels and decreasing mitochondrial damage, thus decreasing autophagosome accumulation.

Our study has some limitations. Firstly, previous evidence has suggested that pan-cathepsins inhibitors could exert anti-tumor effects. However, given that cathepsins have different roles in different tissues, pan-cathepsins inhibitor may exert off-target effects. Moreover, CTSS is characterized as a protease with a broad active range of pH, while other members of cathepsins may not show activity under neutral *in vitro* environment. Other members of cathepsins may exert important role in tumor acidized microenvironment *in vivo*, but our present culture experiments had a disadvantage to prove this. Secondly, though cytosolic calcium change seems important to MCU change, blockade of cytosolic Ca^2+^ could not totally reverse CTSS-inhibition induced effects, including MCU levels change. This suggested that CTSS may have other ways to regulate MCU protein levels. We will clarify this speculation in our further study.

Collectively, our results suggested that knockdown of CTSS contributed to mitochondrial Ca^2+^ overload by upregulating the expression of MCU and impaired mitophagy by blocking autophagic flux in GBC lines, eventually leading to mitochondrial apoptosis.

## Data Availability Statement

The raw data supporting the conclusions of this article will be made available by the authors, without undue reservation.

## Ethics Statement

The studies involving human participants were reviewed and approved by the Institutional Review Board, Nanjing University. The patients/participants provided their written informed consent to participate in this study. The animal study was reviewed and approved by the Institutional Animal Care and Use Committee of Jinling Hospital and Nanjing Medical University.

## Author Contributions

MF, YZ, TT, and WN carried out the samples collection and performed the experiments. HW and LZ revised the manuscript. MF, LZ, HW, YZ, and YH designed the studies. MF and LZ wrote the manuscript. All authors contributed to the article and approved the submitted version.

## Funding

This study was grant-supported by the National Natural Science Foundation of China (No.81672503 and No.81702484).

## Conflict of Interest

The authors declare that the research was conducted in the absence of any commercial or financial relationships that could be construed as a potential conflict of interest.
