# Supplementary material for: Inhibition of Cathepsin S Induces Mitochondrial Apoptosis in Glioblastoma Cell Lines Through Mitochondrial Stress and Autophagosome Accumulation
Source: Front Oncol. 2020 Dec 23;10:516746. doi: 10.3389/fonc.2020.516746 (PMC7787074; doi:10.3389/fonc.2020.516746)
Supplement: Supplementary file 1 [file DataSheet_1.pdf]

Figure 1

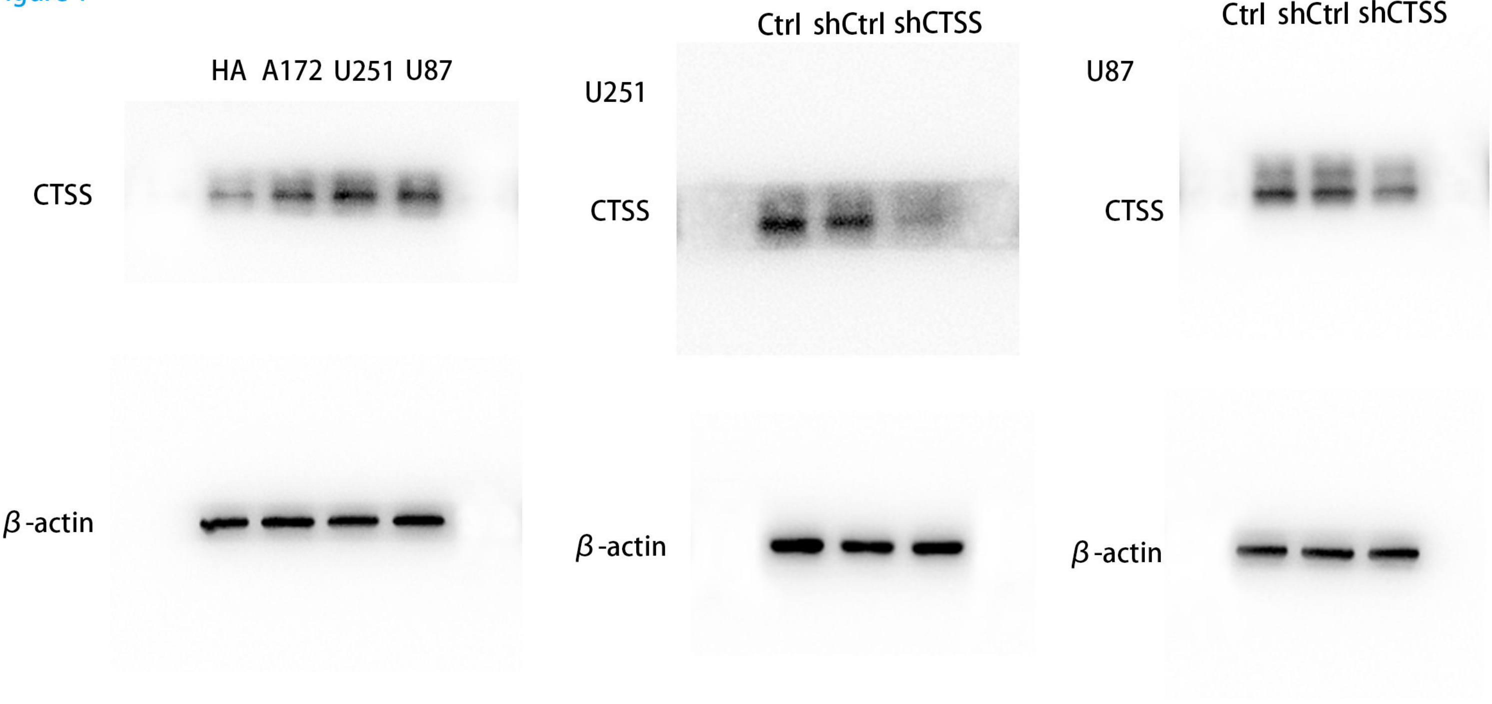

Figure 2

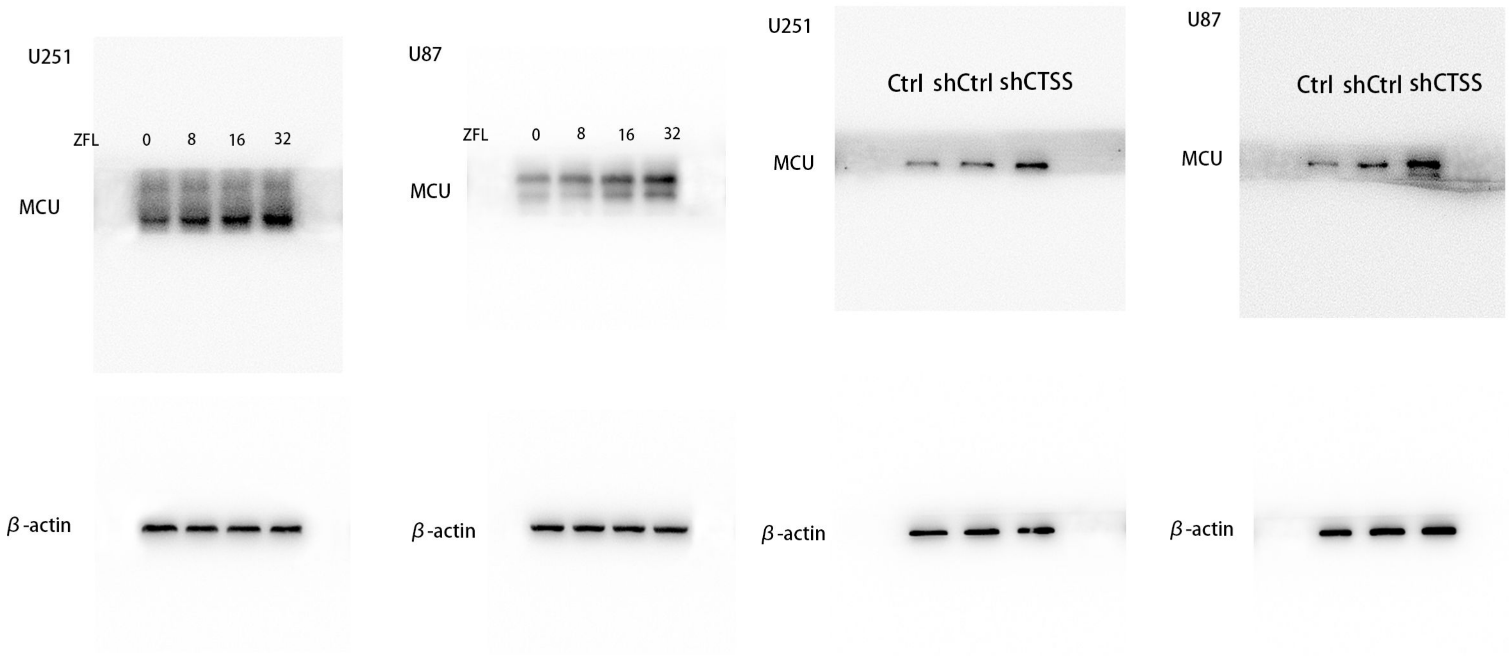

Figure3

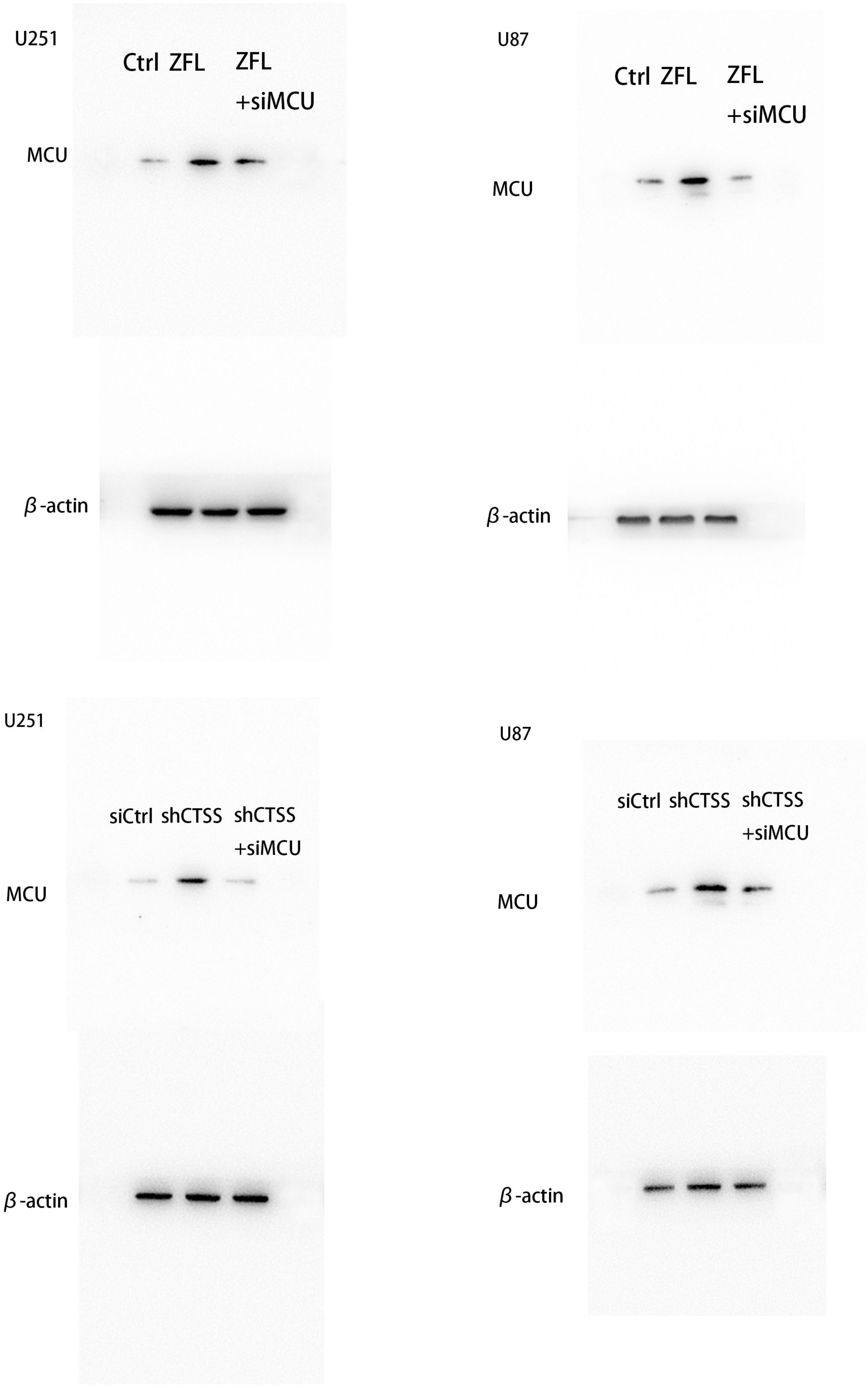

U251
Ctrl
ZFL
ZFL
+siMCU
MCU
β-actin
U87
Ctrl
ZFL
ZFL
+siMCU
MCU
β-actin
U251
siCtrl
shCTSS
shCTSS
+siMCU
MCU
β-actin
U87
siCtrl
shCTSS
shCTSS
+siMCU
MCU
β-actin

Figure 4

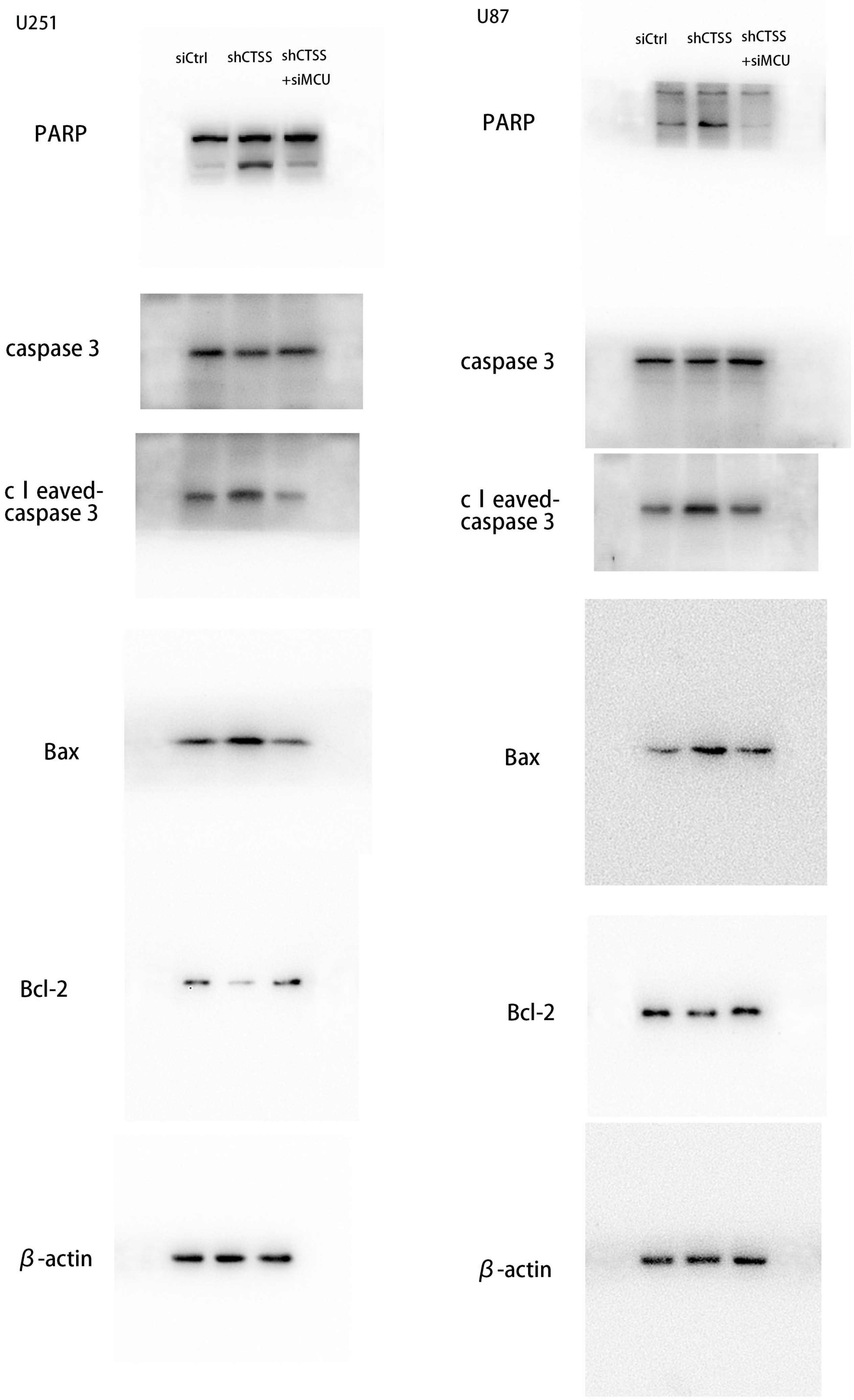
U251
siCtrl
shCTSS
shCTSS
+siMCU
PARP
caspase 3
c l eaved-
caspase 3
Bax
Bcl-2
β-actin
U87
siCtrl
shCTSS
shCTSS
+siMCU
PARP
caspase 3
c l eaved-
caspase 3
Bax
Bcl-2
β-actin

5-1

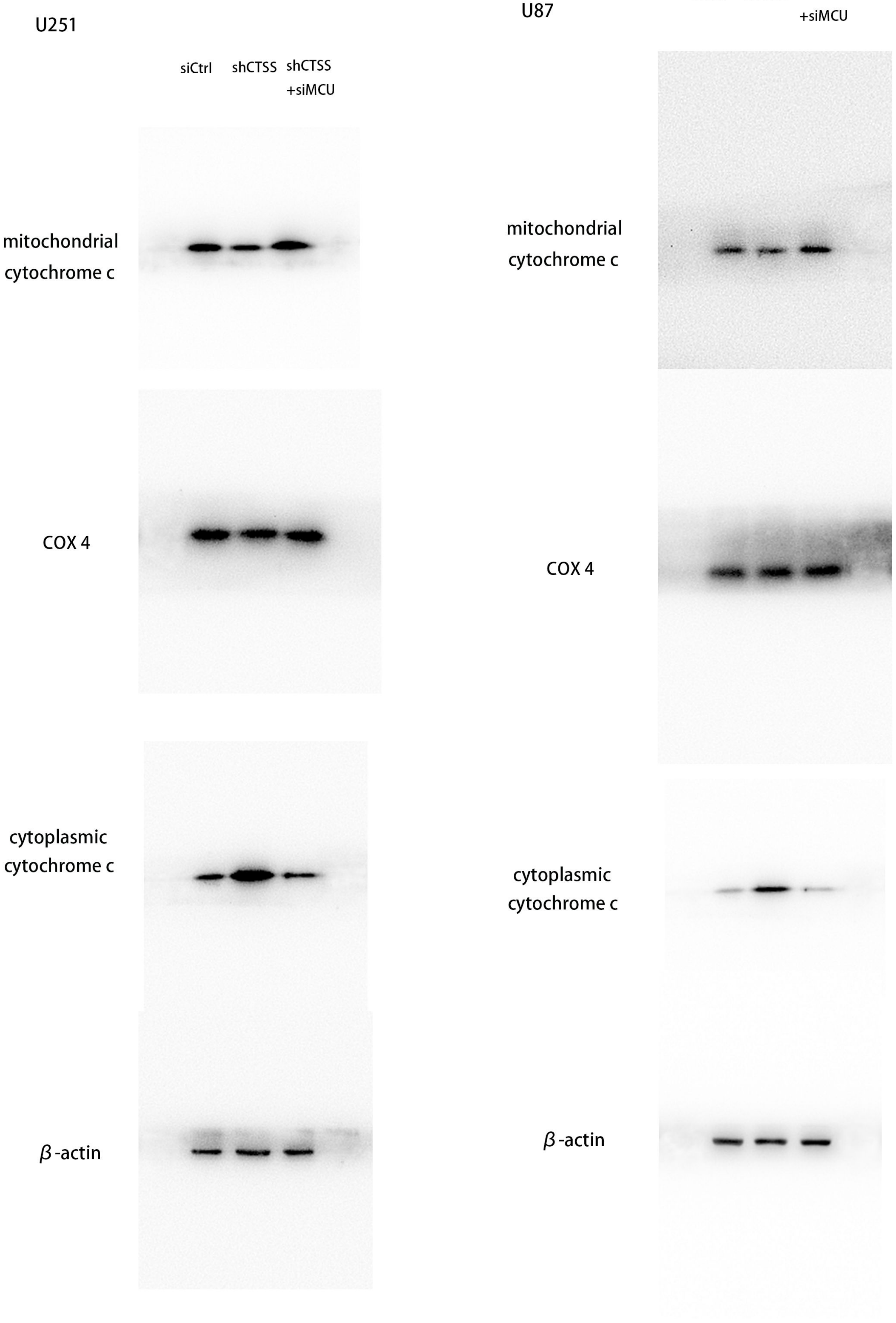

U251
siCtrl
shCTSS
shCTSS +siMCU
mitochondrial cytochrome c
COX 4
cytoplasmic cytochrome c
β-actin
U87
siCtrl
shCTSS
shCTSS +siMCU
mitochondrial cytochrome c
COX 4
cytoplasmic cytochrome c
β-actin

6-1

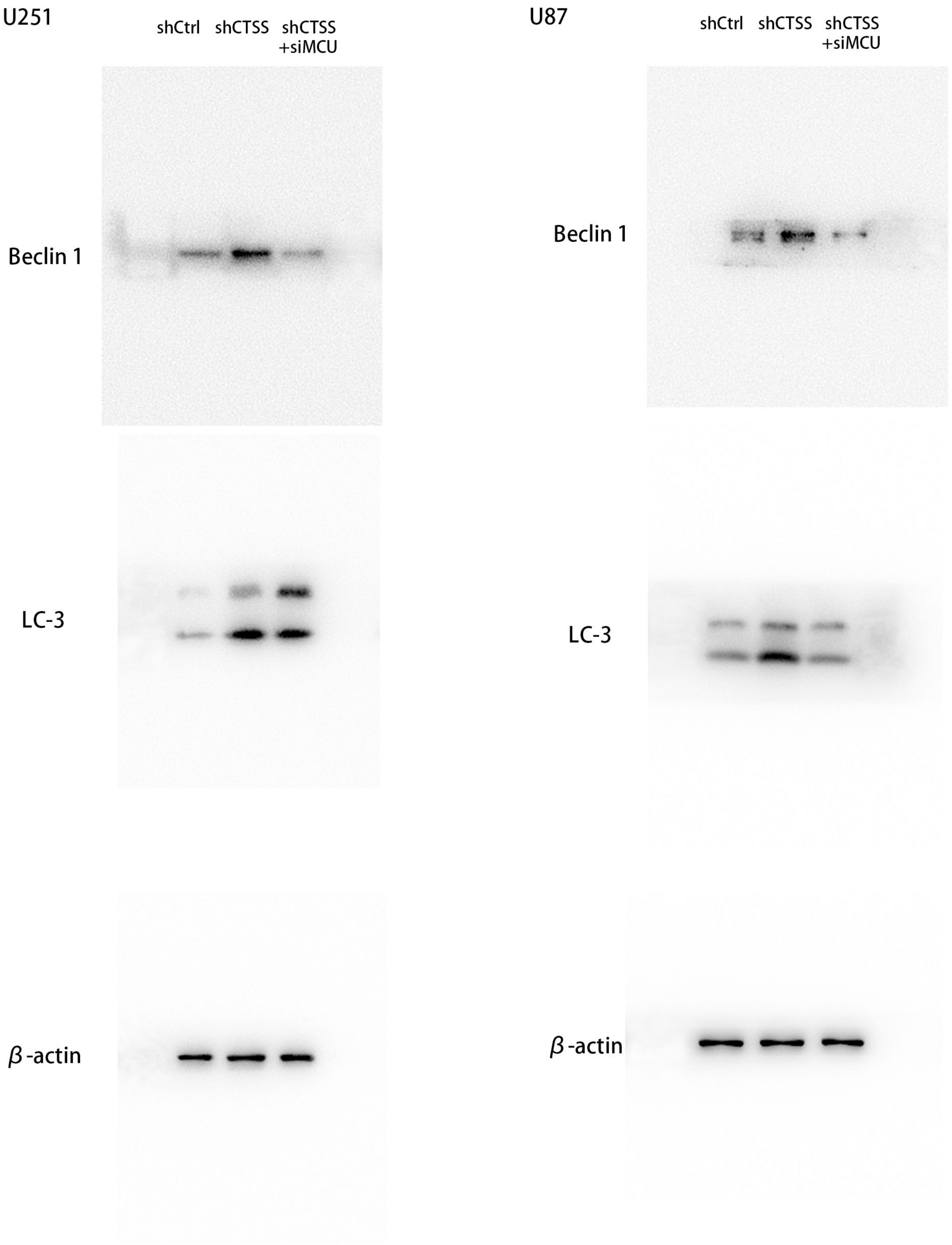
U251
shCtrl
shCTSS
shCTSS +siMCU
Beclin 1
LC-3
β-actin
U87
shCtrl
shCTSS
shCTSS +siMCU
Beclin 1
LC-3
β-actin

6-2

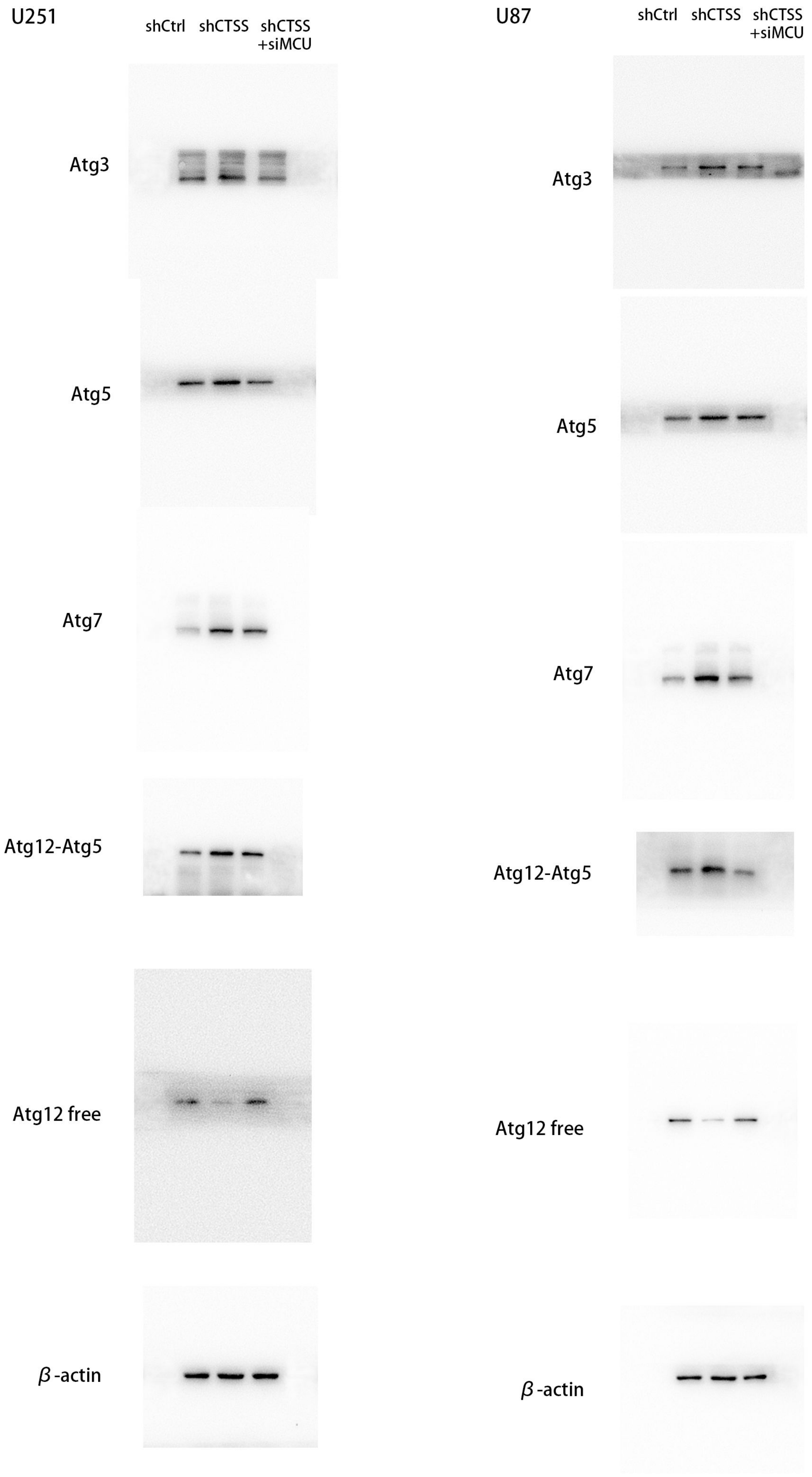
U251
shCtrl
shCTSS
shCTSS +siMCU
Atg3
Atg5
Atg7
Atg12-Atg5
Atg12 free
β-actin
U87
shCtrl
shCTSS
shCTSS +siMCU
Atg3
Atg5
Atg7
Atg12-Atg5
Atg12 free
β-actin

6-3

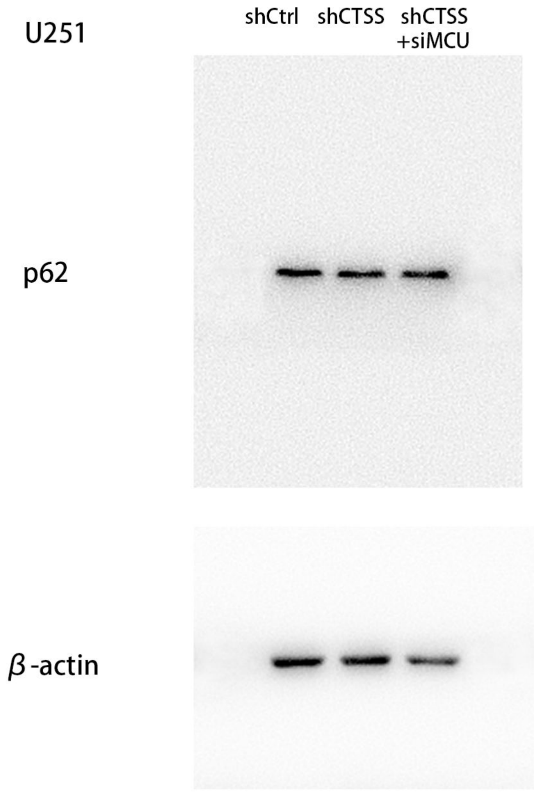
U251
shCtrl
shCTSS
shCTSS
+siMCU
p62
β-actin

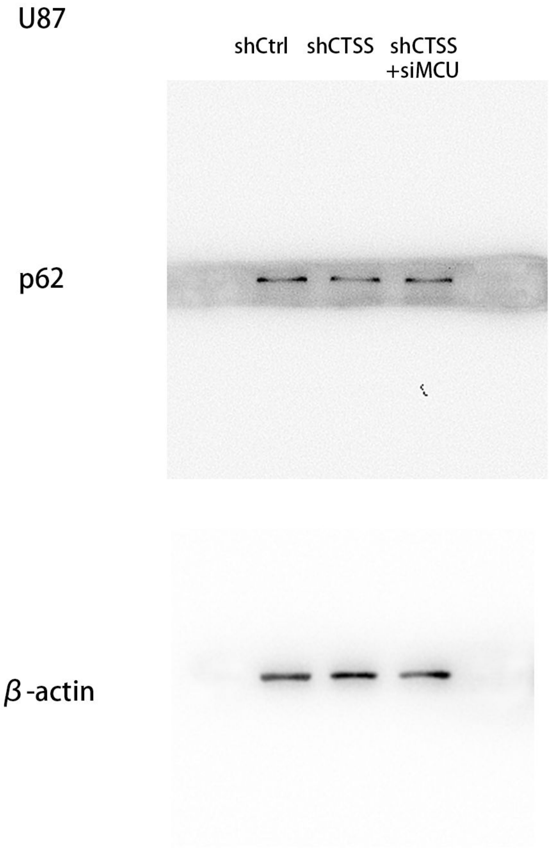
U87
shCtrl
shCTSS
shCTSS
+siMCU
p62
β-actin
