# Supplementary material for: Inhibition of Cathepsin S Induces Mitochondrial Apoptosis in Glioblastoma Cell Lines Through Mitochondrial Stress and Autophagosome Accumulation
Source: Front Oncol. 2020 Dec 23;10:516746. doi: 10.3389/fonc.2020.516746 (PMC7787074; doi:10.3389/fonc.2020.516746)
Supplement: Supplementary file 2 [file DataSheet_2.docx]

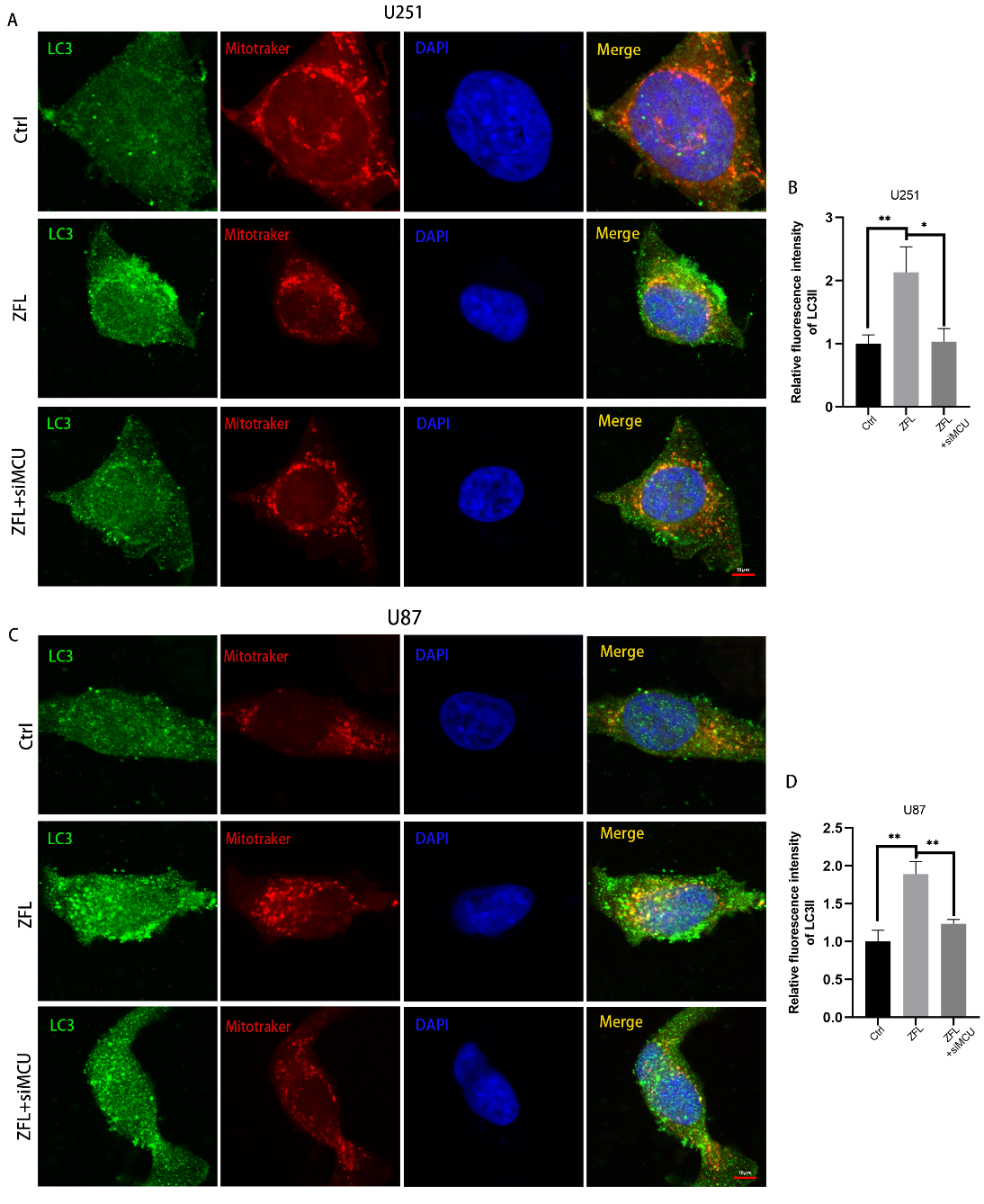


**Supplementary.7** (A-D) After cells were treated as above, mitochondria from different groups were stained with MitoTracker Red. Co-staining for LC3 was conducted as described. More LC3-positive particles were observed in the ZFL-treated groups than in the control groups. Additionally, LC3 was close to the mitochondria in the ZFL-groups. Moreover, silence of MCU partly decreased the proportion of LC3-positive particles. Scale bar, 10 μm. Data are represented as the means ± SEMs of three independent experiments. *p<0.05, **p<0.01 versus the control group.
